# Efficient electroluminescent hybridized local and charge-transfer host materials with small singlet–triplet splitting to enhance exciton utilization efficiency: excited state transition configuration†

**DOI:** 10.1039/c9ra00135b

**Published:** 2019-02-26

**Authors:** Jayaraman Jayabharathi, Venugopal Thanikachalam, Ganapathy Abirama Sundari

**Affiliations:** Department of Chemistry, Annamalai University Annamalainagar-608002 Tamilnadu India jtchalam2005@yahoo.co.in +91 9443940735

## Abstract

A series of efficient electroluminescent materials with dual carrier transport properties shows enhanced singlet exciton utilization (*η*_s_) due to small singlet–triplet splitting (Δ*E*_ST_). The strong orbital-coupling transitions of *N*-(4-(1-(1-(2,3-dihydrobenzo[*b*][1,4]dioxin-6-yl)-4,5-diphenyl-1*H*-imidazol-2-yl)naphthalen-4-yl)phenyl)-*N*-phenyl benzenamine (DDPB) exhibit deep blue emission at 435 nm (CIEy, 0.07) with an external quantum efficiency of 2.01%. The electroluminescent efficiencies of 2-(1-(9*H*-carbazol-9-yl)naphthalen-4-yl)-1-(2,3-dihydrobenzo[*b*][1,4]dioxin-6-yl)-1*H*-phenanthro[9,10-*d*]imidazole (CDDPI) (*L* – 3992 cd m^−2^; *η*_ex_ – 3.01%; *η*_c_ – 2.56 cd A^−1^; *η*_p_ – 2.12 lm W^−1^) are higher than those of the *N*-(4-(1-(1-(2,3-dihydrobenzo[*b*][1,4]dioxin-6-yl)-*H*-phenanthro[9,10-*d*]imidazole-2-yl)naphthalen-4-yl)phenyl)-*N*-phenylbenzenamine (DBDPA) based device (*L* – 3015 cd m^−2^; *η*_ex_ – 2.85%; *η*_c_ – 2.01 cd A^−1^; *η*_p_ – 1.92 lm W^−1^). The blue emissive materials CDDPI and DBDPA are used as a host to construct green and red phosphorescent OLEDs: the green device based on CDDPI:Ir(ppy)_3_ exhibits higher efficiencies (*L* – 8812 cd m^−2^; *η*_ex_ – 19.0%; *η*_c_ – 27.5 cd A^−1^; *η*_p_ – 33.0 lm W^−1^) at 2.7 V and the red device based on CDDPI:Ir(MQ)_2_(acac) exhibits a maximum luminance of 39 661 cd m^−2^ with excellent EL efficiencies [*η*_ex_ – 19.2%; *η*_c_ – 27.9 cd A^−1^; *η*_p_ – 29.2 lm W^−1^; CIE (0.64, 0.34)] compared with those of the DBDPA:Ir(MQ)_2_(acac) based device [*L* – 37 621 cd m^−2^; *η*_ex_ – 18.5%; *η*_c_ – 25.2 cd A^−1^; *η*_p_ – 25.8 lm W^−1^; CIE (0.64, 0.34)].

## Abstract 2

CDDPI:Ir(ppy)_3_ exhibits higher efficiencies: *L* = 8812 cd m^−2^; *η*_ex_ = 19.0%; *η*_c_ = 27.5 cd A^−1^; *η*_p_ = 33.0 lm W^−1^ at 2.7 V.

## Graphical abstract

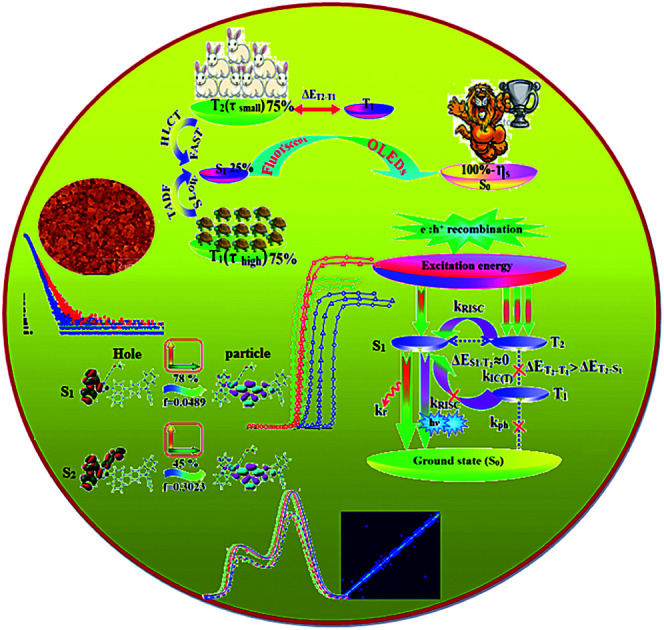

## 1. Introduction

The development of highly efficient blue emissive materials with balanced carrier injection in organic light-emitting devices (OLEDs) remains a challenging task.^1–5^ The blue emitters with higher energy gap (*E*_g_) result in low electron affinities and the lowering of the device efficiency.^6–9^ Though the non-doped blue device based on dipyrenylbenzene (CIE: 0.15, 0.11) and anthracene derivative (CIE: 0.14, 0.12) exhibit high external quantum efficiencies (*η*_ex_) of 5.2% and 5.3%, respectively,^10,11^ their power (*η*_p_) and current (*η*_c_) efficiencies are low due to the wide band-gap (*E*_g_). The blue device (CIE: 0.15, 0.16) with oligoquinoline emissive material shows high *η*_p_ and *η*_ex_ values of 4.3 lm W^−1^ and 6.6%, respectively.^12^ The simple structured device based on bis(phenanthroimidazolyl)biphenyl derivative exhibits higher performances (CIEy-0.15; *η*_ex_: 6.31%) than the multi-layered one.^13,14^ Electroluminescent materials with thermally activated delayed fluorescence (TADF),^15^ hybridized local and charge-transfer (HLCT)^16^ and triplet–triplet annihilation (TTA: *η*_int_ – 62.5%)^17–20^ have enhanced the internal quantum efficiency (*η*_int_).^21–25^ Constructing donor–spacer–acceptor (D–π–A) molecules is one of the widely used techniques to improve the charge injection and carrier transportation of OLED materials.^26^ In some D–π–A based OLEDs, the triplet excitons have been fully employed and excellent performances were obtained.^27,28^ D–π–A compounds usually possess weakly bound charge-transfer (CT) excitons which facilitate a reverse intersystem crossing (RISC) process in the OLEDs. However, it is rather rarely reported that the fluorescent OLEDs based on D–π–A molecular architecture exhibit high efficiency and favourable colour-purity. Hence, the molecular design is aimed to overcome the following remarks: (i) the D–π–A molecules are more suitable to design narrow-band-gap materials due to significant decrease in excited state energy of the CT state from donor to acceptor or more delocalized π–π* state between weak donor and weak acceptor;^26*b*,29,30^ (ii) CT state as an emissive state always leads to broadened photoluminescence (PL) and electroluminescence (EL) spectra which is unfavourable for high colour-purity;^28^ (iii) the CT state usually exhibits a low efficiency fluorescence which is attributed to the nature of the forbidden transition induced by the spatial separation between the hole and electron wave functions. On the contrary, the locally excited (LE) state is suited to producing high-efficiency fluorescence radiation due to the large orbital overlap. However, the CT state can provide a RISC channel that improves exciton-utilizing efficiency (*η*_s_) in fluorescent OLEDs through a very small energy splitting between singlet and triplet states which has been proven to be an effective way to utilize triplet exciton energy in fluorescent OLEDs.^27,28^ Considering the above issues, if CT and LE states could be reasonably combined into one D–π–A compound, it would be possible to further greatly improve the efficiency of fluorescent OLEDs. That is to say, the low-lying LE state determines the efficient fluorescence radiation, wide band-gap and colour-purity, while the high-lying CT state is responsible for the triplet exciton utilization through the RISC process. Thus, this golden combination is surely beneficial to maximize the EL efficiency of OLEDs, and it can be a novel strategy to design emitters with high efficiency (*η*_PL_ and high *η*_s_) and good colour-purity by taking advantage of the D–A structure.

The donor–spacer–acceptor (D–π–A) compounds with HLCT emissive state exhibit high exciton utilization efficiency (*η*_S_) attributed by hot exciton mechanism.^31–33^ The external quantum efficiency (*η*_EQE_) and exciton utilization efficiency (*η*_S_) have been calculated by: *η*_EQE_ = *η*_out_ × *η*_IQE_ = *η*_out_ × *η*_rec_ × *η*_*γ*_ × *Φ*_PL;_*η*_s_ = *η*_out_ × *η*_rec_ × *η*_PL_, respectively.^34^ The LE dominated HLCT provides high radiative rate (*k*_r_) which results high *η*_PL_ whereas the CT dominated HLCT is responsible for small singlet–triplet energy splitting (Δ*E*_ST_).^35–37^ Designing D–π–A molecules is an effective strategy for balancing the carrier transport in the device due to their bipolar ability,^38^ however, the assembly of donor and acceptor units extends the π-conjugation and it is not beneficial for blue emission. Thus, it is difficult to achieve deep-blue emission with CIEy of 0.06: reduced conjugation,^39,40^ twisted conformation^41^ and linkage modification^42^ are the strategies adopted to achieve blue emission. Hence, it is aimed to design D–π–A geometry with side capping of bulky dihydrobenzodioxin in phenanthrimidazole core; expected to enhance the color purity, thermal stability, high quantum efficiency, bipolar properties and high exciton utilizing efficiency.^43–53^ A blue emissive material with balanced carrier transport characteristics and high triplet energy (*E*_T_) may be employed as host for green and red phosphorescent emitters.^54^ The efficient host for green and red phosphors exhibit low efficiency when they used as blue OLEDs.^55^ Therefore, it is still a challenging task to achieve efficient full color OLEDs with blue emissive material. Inspired by this, herein, we report multi-functional organic OLED materials namely, *N*-(4-(1-(1-(2,3-dihydrobenzo[*b*][1,4]dioxin-6-yl)-4,5-diphenyl-1*H*-imidazol-2-yl)naphthalen-4-yl)phenyl)-*N*-phenylbenzenamine (DDBP) obtained by single bond fission of phenanthrene moiety of *N*-(4-(1-(1-(2,3-dihydrobenzo[*b*][1,4]dioxin-6-yl)-*H*-phenanthro[9,10-*d*]imidazole-2-yl)naphthalen-4-yl)phenyl)-*N*-phenylbenzenamine (DBDPA) and 2-(1-(9*H*-carbazol-9-yl)naphthalen-4-yl)-1-(2,3-dihydrobenzo[*b*][1,4]dioxin-6-yl)-1*H*-phenanthro[9,10-*d*]imidazole (CDDPI) used as (i) emitters in blue OLEDs and (ii) host for green and red OLEDs. The strong orbital-coupling transitions of DDPB exhibit deep blue emission at 435 nm with CIEy 0.07 and maximum external quantum efficiency of 2.01%. The electroluminescent efficiencies of CDDPI (*L* – 3992 cd m^−2^; *η*_ex_ – 3.01%; *η*_c_ – 2.56 cd A^−1^; *η*_p_ – 2.12 lm W^−1^) are higher than DBDPA based device (*L* – 3015 cd m^−2^; *η*_ex_ – 2.85%; *η*_c_ – 2.01 cd A^−1^; *η*_p_ – 1.92 lm W^−1^). The blue emissive materials, CDDPI and DBDPA are used as a host to construct green and red phosphorescent OLEDs. The green device with CDDPI:Ir(ppy)_3_ exhibits maximum luminance of 8812 cd m^−2^, maximum *η*_c_ and *η*_p_ of 27.5 cd A^−1^ and 33.0 lm W^−1^, respectively and red device based on CDDPI:Ir(MQ)_2_(acac) exhibits excellent EL efficiencies [*L* – 39 661 cd m^−2^;*η*_ex_ – 19.2%; *η*_c_ – 27.9 cd A^−1^; *η*_p_ – 29.2 lm W^−1^; CIE (0.64, 0.34)].

## 2. Experimental

### 2.1. Synthesis of host dihydrobenzodioxin phenanthroimidazoles

**Scheme 1:**
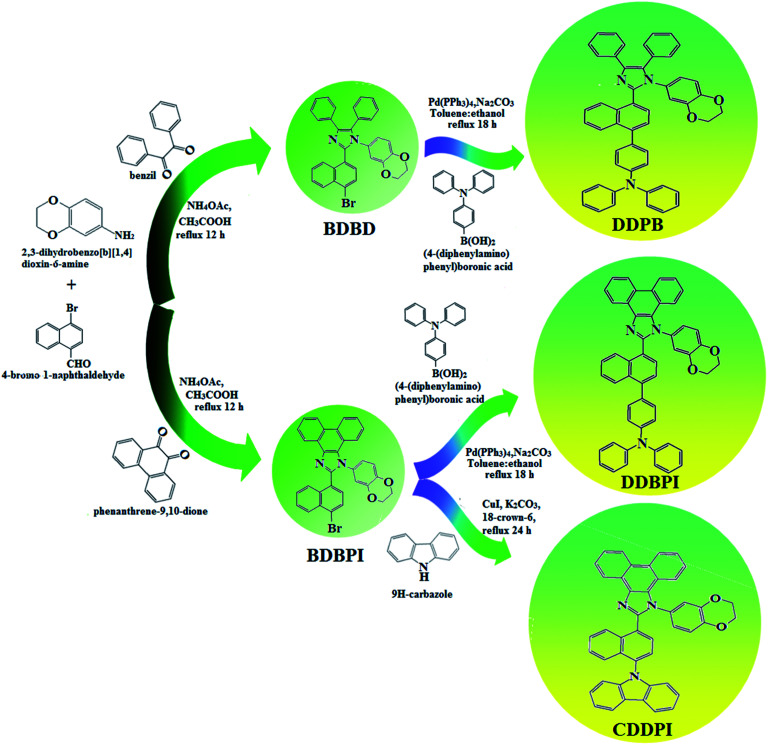
Synthetic routes for BDBD, BDBPI, DDPB, DBDPA and CDDPI.

The schematic synthetic route for the emissive and or host materials namely, *N*-(4-(1-(1-(2,3-dihydrobenzo[*b*][1,4]dioxin-6-yl)-4,5-diphenyl-1*H*-imidazol-2-yl)naphthalen-4-yl)phenyl)-*N*-phenylbenzenamine (DDPB), *N*-(4-(1-(1-(2,3-dihydrobenzo[*b*][1,4]dioxin-6-yl)-*H*-phenanthro[9,10-*d*]imidazole-2-yl)naphthalen-4-yl)phenyl)-*N*-phenylbenzenamine (DBDPA) and 2-(1-(9*H*-carbazol-9-yl)naphthalen-4-yl)-1-(2,3-dihydrobenzo[*b*][1,4]dioxin-6-yl)-1*H*-phenanthro[9,10-*d*]imidazole (CDDPI) are displayed in Scheme 1. Benzil (1 mmol) for BDBD or phenanthrene-9,10-dione (1 mmol) for BDBPI, 4-bromo-1-naphthaldehyde (1 mmol), 1,4-benzodioxane-6-amine (1 mmol) and ammonium acetate (1 mmol), all in 25 mL acetic acid was refluxed (120 °C; 24 h) and the crude 2-(1-bromonaphthalen-4-yl)-1-(2,3-dihydrobenzo[*b*][1,4]dioxin-6-yl)-4,5-diphenyl-1*H*-imidazole (BDBD) or 2-(1-bromonaphthalen-4-yl)-1-(2,3-dihydrobenzo[*b*][1,4]dioxin-6-yl)-1*H*-phenanthro[9,10-*d*]imidazole (BDBPI) was column chromatographed (hexane : ethyl acetate) and the pure sample was used for the synthesis of DDPB, DBDPA and CDDPI, respectively.

#### 2.1.1. *N*-(4-(1-(1-(2,3-Dihydrobenzo[*b*][1,4]dioxin-6-yl)-4,5-diphenyl-1*H*-imidazol-2-yl)naphthalen-4-yl)phenyl)-*N*-phenylbenzenamine (DDPB)

2-(1-Bromonaphthalen-4-yl)-1-(2,3-dihydrobenzo[*b*][1,4]dioxin-6-yl)-4,5-diphenyl-1*H*-imidazole (BDBD) (4.5 mmol), Pd(PPh_3_)_4_ (0.25 mmol) and 4-(diphenylamino)phenylboronic acid (7.5 mmol), all in aqueous Na_2_CO_3_ (15 mL) in toluene : ethanol (20 : 15 mL) was refluxed under N_2_ stream for 24 h. The dichloromethane extract was distilled off to get white DDPB. Yield 60%. Anal. calcd: C_51_H_37_N_3_O_2_: C, 84.62; H, 5.15; N, 5.81. Found: C, 84.59; H, 5.10; N, 5.77. 400 MHz ^1^H NMR (CDCl_3_): *δ* 4.35–4.38 (m, 4H), 6.46 (d, *J* = 8.2 Hz, 2H), 6.62–6.70 (m, 9H), 7.01 (t, 4H), 7.23–7.32 (m, 10H), 7.48 (d, *J* = 8.4 Hz, 4H), 7.60–7.67 (m, 4H) (Fig. S1†). 100 MHz ^13^C NMR (CDCl_3_): *δ* 64.21, 101.52, 114.71, 115.87, 122.62, 122.82, 125.32, 127.54, 128.24, 128.86, 131.08, 131.84, 133.15, 133.28, 134.86, 136.58, 141.45, 144.65, 147.61 (Fig. S2†). MALDI TOF MS: *m*/*z*. 723.29 [M^+^] (Fig. S7†). Calcd: 723.25.

#### 2.1.2. *N*-(4-(1-(1-(2,3-Dihydrobenzo[*b*][1,4]dioxin-6-yl)-*H*-phenanthro[9,10-*d*]imidazole-2-yl)naphthalen-4-yl)phenyl)-*N*-phenylbenzenamine (DBDPA)

2-(1-Bromonaphthalen-4-yl)-1-(2,3-dihydrobenzo[*b*][1,4]dioxin-6-yl)-1*H*-phenanthro[9,10-d]imidazole (BDBPI) (4.5 mmol), Pd(PPh_3_)_4_ (0.25 mmol), 4-(diphenylamino) phenylboronic acid (7.5 mmol) and aqueous Na_2_CO_3_ (15 mL), all in toluene : ethanol (20 : 15 mL) was refluxed for 2 days under N_2_ stream. The dichloromethane extract was distilled off to get DBDPA. Yield 52%. Anal. calcd: C_51_H_35_N_3_O_2_: C, 84.86; H, 4.89; N, 5.82. Found: C, 84.83; H, 4.87; N, 5.80. 400 MHz ^1^H NMR (CDCl_3_): *δ* 4.36–4.39 (m, 4H), 6.46 (d, *J* = 8.8 Hz, 2H), 6.61–6.69 (m, 9H), 7.12 (t, 4H), 7.24–7.34 (m, 4H), 7.61 (d, *J* = 8.8 Hz, 4H), 7.82–7.88 (m, 4H), 8.12 (d, *J* = 8.4 Hz, 2H), 8.93 (d, *J* = 8.0 Hz, 2H) (Fig. S3†). 100 MHz ^13^C NMR (CDCl_3_): *δ* 64.41, 101.70, 114.81, 115.86, 122.35, 122.61, 125.29, 126.41, 126.54, 126.72, 127.64, 128.41, 129.76, 129.81, 131.54, 133.45, 134.98, 136.86, 140.08, 141.25, 147.75, 149.51 (Fig. S4†). MALDI TOF MS: *m*/*z*. 721.27 [M^+^] (Fig. S7†). Calcd: 721.21.

#### 2.1.3. 2-(1-(9*H*-Carbazol-9-yl)naphthalen-4-yl)-1-(2,3-dihydrobenzo[*b*][1,4]dioxin-6-yl)-1*H*-phenanthro[9,10-*d*]imidazole (CDDPI)

A mixture of 2-(1-bromonaphthalen-4-yl)-1-(2,3-dihydrobenzo[*b*][1,4]dioxin-6-yl)-1*H*-phenanthro[9,10-*d*]imidazole (BDBPI) (4.5 mmol), 9*H*-carbazole (7.5 mmol), CuI (10.0 mg, 0.05 mmol), 18-crown-6 (13.2 mg, 0.05 mmol), and K_2_CO_3_ (0.83 g, 6.0 mmol) in tetrahydro-1,3-dimethylpyrimidin-2(1*H*)-one (2.0 mL) was refluxed in nitrogen atmosphere for 18 h. The solvent was distilled off and the pure CDDPI was used further analysis. Yield 58%. Mp 305 °C. Anal. calcd: C_45_H_29_N_3_O_2_: C, 83.96; H, 4.54; N, 6.53. Found: C, 83.82; H, 4.47; N, 6.40. 400 MHz ^1^H NMR (CDCl_3_): *δ* 4.41–4.50 (m, 4H), 6.16 (d, *J* = 8.2 Hz, 3H), 6.62–6.70 (m, 6H), 7.14 (t, 4H), 7.22–7.36 (m, 4H), 7.68 (d, *J* = 8.8 Hz, 4H), 8.22 (d, *J* = 8.4 Hz, 2H), 8.82 (d, *J* = 8.0 Hz, 2H) (Fig. S5†). 100 MHz ^13^C NMR (CDCl_3_): *δ* 64.25, 101.61, 111.15, 114.71, 115.88, 119.51, 120.35, 121.21, 122.25, 123.51, 125.54, 126.72, 126.84, 127.65, 128.31, 129.76, 130.51, 131.54, 132.85, 133.98, 135.86, 139.56, 146.08, 147.25, 149.46 (Fig. S6†). MALDI TOF MS: *m*/*z*. 643.17 [M^+^] (Fig. S7†). Calcd: 643.23.

### 2.2. Measurements and general methods

All reagents used for designing the manuscript are purchased from Sigma-Aldrich and NMR was recorded with 400 MHz spectrometer (Bruker). Agilent (LCMS VL SD) spectrometry was employed to analyze the mass of the blue emitters and or host materials. UV-vis absorption was measured on a Perkin-Elmer Lambda 35 (solution) and Lambda 35 spectrophotometer with integrated sphere (RSA-PE-20) instrument (film). PerkinElmer LS55 fluorescence spectrometer was employed to analyze the emission properties. Thermogravimetric analysis (TGA) and differential scanning calorimetric (DSC) were recorded with PerkinElmer thermal analysis system and NETZSCH-DSC-204, respectively. Time correlated single photon counting (TCSPC) spectrometer (Horiba Fluorocube-01-NL lifetime system and nano LED is excitation source with TBX-PS is detector: DAS6 software and *χ*^2^ – 0.8–1.2) was employed to examine the decay time of the emitters. The absolute PLQY was determined with fluorescence spectrometer Model-F7100. Cyclic voltammetry (CV) was recorded with potentiostat CHI 630A electrochemical analyzer with 100 mV s^−1^ scan (Ag/Ag^+^-reference electrode, platinum electrode-working electrode and platinum wire-counter electrode, ferrocene-internal standard HOMO-4.80 eV and 0.1 M tetrabutylammonium perchlorate-supporting electrolyte) and the HOMO [*E*_HOMO_ = −(*E*_ox_ + 4.8 eV)] and LUMO energies [*E*_LUMO_ = *E*_HOMO_ − 1239/*λ*_onset_] of the blue emitters were calculated.

### 2.3. Computational details

For theoretical calculation, ground state (DFT)/excited state (TD-DFT) geometrical properties were optimized by employing Gaussian 09 program.^56^ Multifunctional wavefunction analyzer (Multiwfn)^57^ was used to know the nature of electronic transition of excited states and natural transition orbitals (NTOs).

### 2.4. Fabrication of devices

#### 2.4.1. Device fabrication and measurement

Devices with configuration of (i) [ITO/NPB (70 nm)/DDPB/DBDPA/CDDPI (100 nm)/TPBI (20 nm)/LiF (0.5 nm)/Al], (ii) hole-only device: [ITO/HATCN (10 nm)/NPB (20 nm)/DBDPA/CDDPI (60 nm)/NPB (20 nm)/Al (100 nm)], (iii) electron-only device: [ITO/TPBi (10 nm)/DBDPA/CDDPI (60 nm)/TPBi (10 nm)/LiF (1 nm)/Al (100 nm)], (iv) green device: [ITO/NPB (40 nm)/TCTA (5 nm)/DBDPA (30 nm): 5 wt% Ir(ppy)_3_/CDDPI (30 nm): 5 wt% Ir(ppy)_3_/TPBI (50 nm)/LiF (1 nm)/Al (100 nm)] and (v) red device: [ITO/NPB (40 nm)/TCTA (5 nm)/DBDPA (30 nm): 8 wt% Ir(MQ)_2_(acac)/CDDPI (30 nm): 8 wt% Ir(MQ)_2_(acac)/TPBI (50 nm)/LiF (1 nm)/Al (100 nm)] were fabricated on pre-cleaned ITO-coated glass substrates with resistance of 20 Ω sq^−1^. Current density–voltage characteristics were measured with Keithley 2400 power source. The EL spectra and CIE coordinates were recorded with spectrometer (USB-650-VIS-NIR, Ocean Optics, Inc, USA).

## 3. Results and discussion

### 3.1. Potential energy scan (PES) and HLCT character

**Fig. 1:**
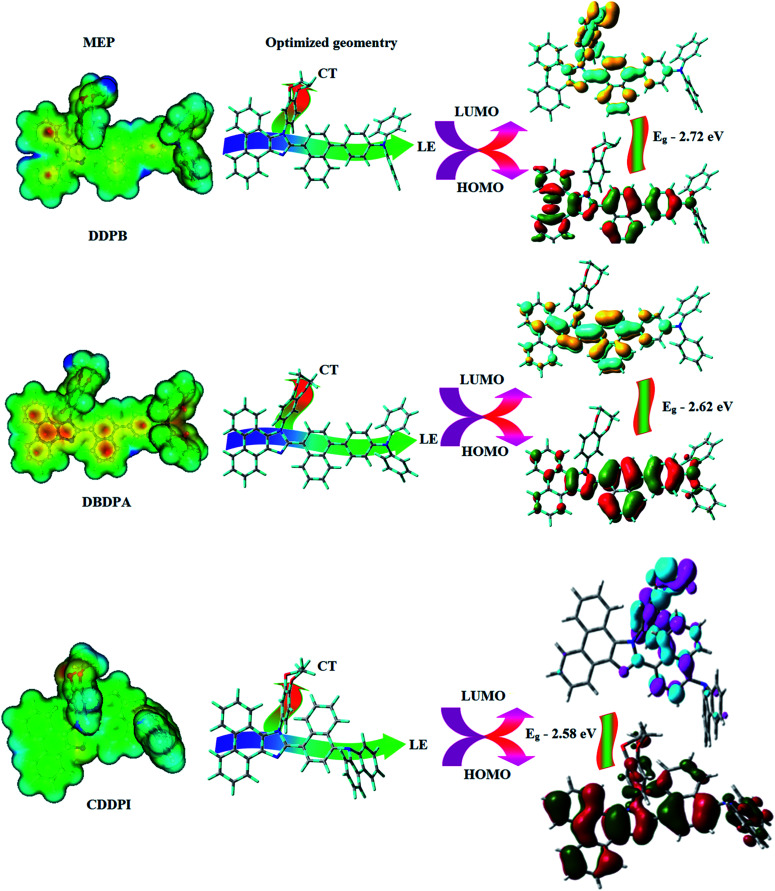
HOMO, LUMO contour maps and molecular electrostatic potential (ESP) surface of DDPB, DBDPA and CDDPI.

**Fig. 2:**
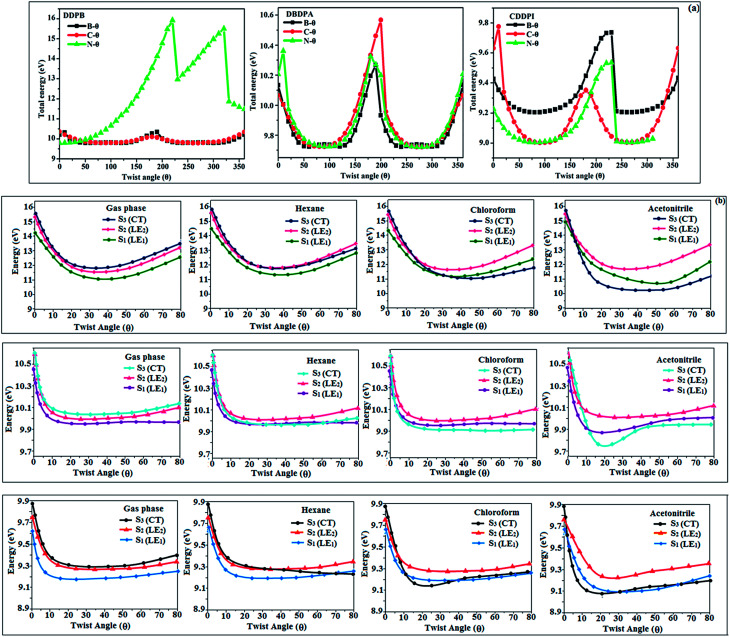
(a) Potential energy scan (PES) at different twist angles C–*θ*, B–*θ* and N–*θ* of DDPB, DBDPA and CDDPI; (b) potential energy scan (PES) of excited states of DDPB, DBDPA and CDDPI with increasing solvent polarity.

The ground state (S_0_) and excited state (S_1_) geometries of D–π–A compounds, DBDPA, CDDPI and DDPB were optimized with DFT/B3LYP/6-31G(d,p) and TD-DFT/B3LYP/6-31G(d,p) methods using Gaussian-09 (Fig. 1). The non-coplanar geometry of DDPB, DBDPA and CDDPI was confirmed by DFT studies. The naphthyl linkage adopts planar geometry and the dihydrobenzodioxin at imidazole nitrogen is perpendicular about 77.6° (DDPB), 87° (DBDPA) and 80° (CDDPI) with respect to their corresponding imidazole plane due to the interaction between aryl rings and repulsion between adjacent hydrogen atoms. Theoretical studies confirmed that DDPB exist in fully twisting geometry whereas DBDPA and CDDPI exist in partially twisting molecular structure, respectively with rigid phenanthro[9,10-*d*]imidazole plane.^58^ The intramolecular charge migration was detailed by potential energy surface scan of DDPB, DBDPA and CDDPI. The twist angles (*θ*°), namely, (i) C–*θ*° (*θ*_1_) (between phenanthrimidazole plane and naphthyl at imidazole carbon), (ii) N–*θ*° (*θ*_2_) (between phenanthrimidazole plane and dihydrobenzodioxin at imidazole nitrogen) and (iii) NA–*θ*° (*θ*_3_) (between naphthyl and phenyl group TPA for DDPB and DBDPA : naphthyl and carbazole for DDPB) imparts a major role in HOMO–LUMO π-electron overlap (Fig. 2).^58^ Because of the steric interaction of neighboring hydrogen atoms, the dihydrobenzodioxin at imidazole nitrogen is perpendicular to imidazole fragment and the conjugation was restricted. Because of the stronger repulsion between the neighboring hydrogen atoms of phenyl ring in DBDPA and CDDPI, the ground state twist angle (*θ*_1_) of DBDPA (88°) and CDDPI (73°) is higher when compared with DDPB (62°), however, increased excited state twist angle (*θ*_1_) of DDPB (108°), DBDPA (98°) and CDDPI (82°) was obtained.^59^

The TPA moiety in DDPB and DBDPA and Cz moiety in CDDPI was twisted with dihedral angle of 130.8°, 148.2° and 159.1°, respectively.^60^ The larger twist angle of CDDPI when compared with DDPB and DBDPA is due to the stronger repulsion between the two adjacent hydrogen atoms in carbazole and naphthyl spacer as a result of stronger rigidity of Cz than TPA. The excited state twist angle (*θ*_2_) of DDPB, DBDPA and CDDPI are increased to 79.1°, 89.0° and 91.0°, respectively when compared with ground state twist angle (*θ*_2_). Similarly smaller increased twist angle (*θ*_1_) was obtained for CDDPI (173°) when compared with DDPB (108.1°) and DBDPA (98.1°). The elongated bond length (*R*_1_) of DDPB (0.06), DBDPA (0.03 Å) and CDDPI (0.02 Å) was calculated from S_0_ to S_1_. The smaller change in geometry of Cz in CDDPI from ground state (S_0_) to excited state (S_1_) was observed than that of TPA unit in DBDPA. This may facilitate the suppression of non-radiation for the enhancement of *η*_PL_.^60^ From the potential energy surfaces of the twisted geometry of CDDPI at ground and excimer at excited states (Fig. 2) reveal that the CDDPI needs very small relaxation energy to form excimer of excited state corresponding to slightly increased interplanar separation of carbazole moieties from the linker fragment (1.4680 Å to 1.4880 Å, 36.6° to 38.6°, Fig. S8†) and then only small energy needs to return to an equilibrium geometry at ground state. At this point, the CDDPI with rigid geometry from initial state to excimer indicating that the minimized non-radiative energy dissipation contributes to the enhanced emission. Hence, the lifetime of excitons are increased due to the suppression of non-radiative pathways. Since the non-radiative pathway of CDDPI was blocked effectively, CDDPI shows higher photoluminescence efficiency (*η*_PL_).^61^ The molecule shows twisted structure which is essential for separating HOMO and LUMO distribution effectively which can also achieve fully excition utilization (*η*_s_) through efficient upconversion of non-radiative triplets to radiative singlets. The overlap between HOMO and LUMO is extremely small for CDDPI, ensuring the small Δ*E*_ST_ of the molecule. Meanwhile, the slight HOMO–LUMO overlap also ensures fairish radiative decay from intramolecular CT excitons. Moreover, large steric hindrances of carbazole group, naphthyl and phenanthrimidazole core lead to a rigid structure of CDDPI compound, which can limit torsional flexibility, thus preventing non-radiative transition^62^

This orthogonal dihedral angle confirmed the non-coplanar twisting conformation of DDPB, DBDPA and DBDPA which suppresses the red shift and harvested high external quantum efficiency (*η*_ex_) in film by restraining intermolecular interaction.^63–66^Fig. 2 shows that the relative energy of DDPB is higher than that of DBDPA and CDDPI. The twist angles (*θ*_2_ and *θ*_3_) in D-A linkage of DDPB, DBDPA and CDDPI could be the origin for CT and LE intercross. In DDPB, DBDPA and CDDPI, the S_1_ state remained unchanged with increasing solvent polarity whereas the S_3_ state decreased to intercross with S_1_ state at moderate polarity and to be much lower than S_1_ state at high polarity (Fig. 2).

### 3.2. Thermal properties and electrochemical properties

**Fig. 3:**
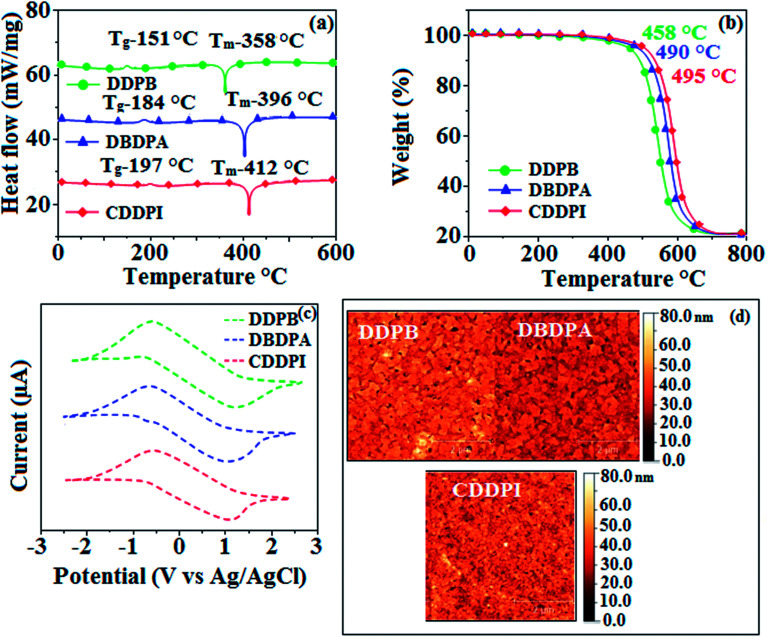
(a) DSC graph; (b) TGA graph (c) cyclic voltammogram and (d) AFM images of DDPB, DBDPA and CDDPI.

**Fig. 4:**
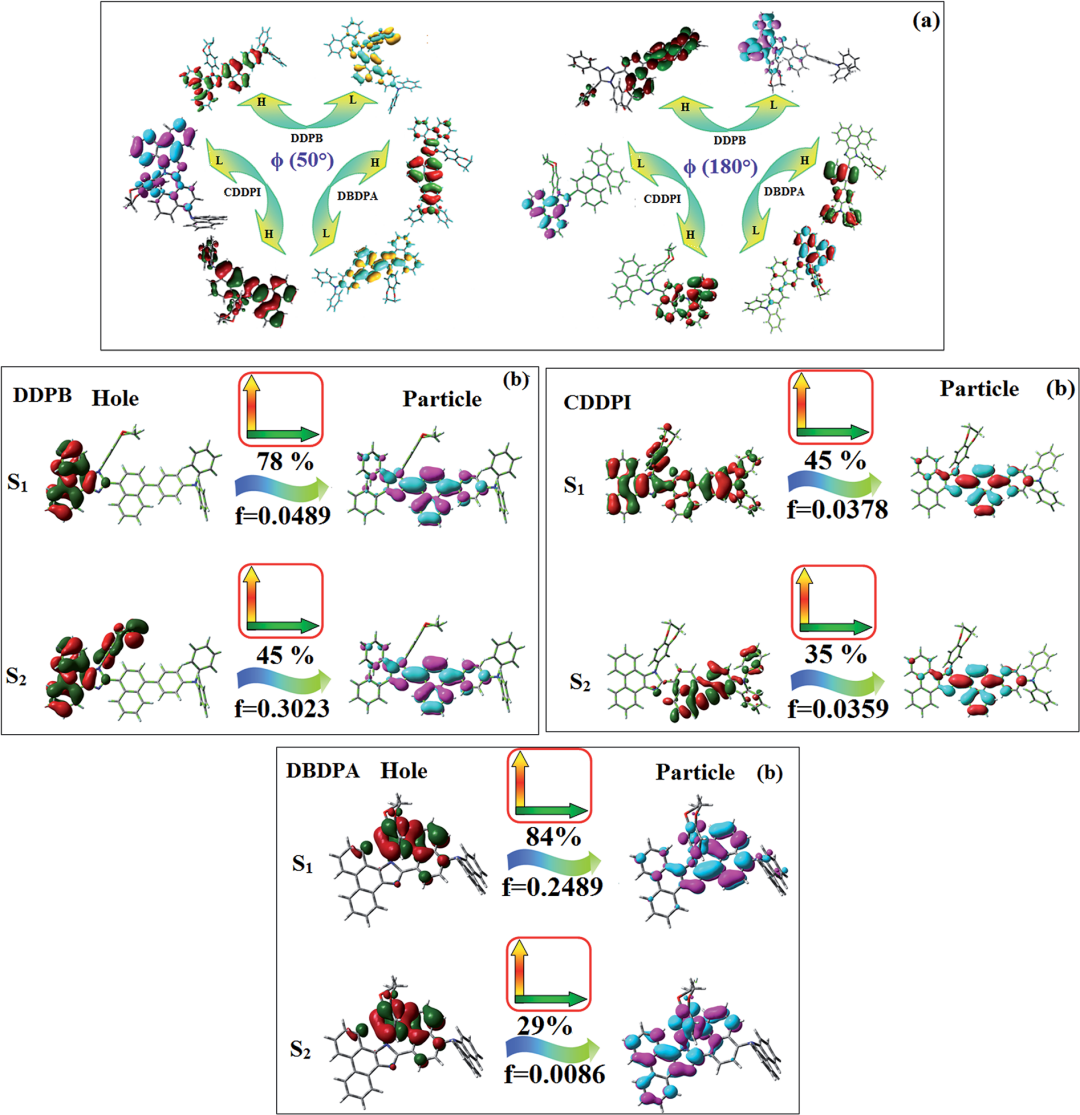
(a) Frontier molecular orbitals of DDPB, DBDPA and CDDPI at 50° and 180° twist angles; (b) NTOs squint of DDPB, DBDPA and CDDPI.

The incorporation of highly rigid bulky moiety at imidazole carbon and side capping at imidazole nitrogen enlarged the size and improved their thermal stability (*T*_d5_ & *T*_g_) which is required for efficient devices (Table 1). Among the donor–spacer–acceptor compounds, DDPB (*T*_g_ – 151 and *T*_d5_ – 458 °C), DBDPA (*T*_g_ – 184 and *T*_d5_ – 490 °C) and CDDPI (*T*_g_ – 197 and *T*_d5_ – 495 °C), CDDPI exhibit high glass transition temperature (*T*_g_) and thermal decomposition temperature (*T*_d5_) (Fig. 3). The higher thermal stability of CDDPI is because of the stronger rigidity of Cz than TPA which will be in favor of OLED stability.^66–69^ The improved *T*_g_ was probably due to the intermolecular interaction which can induce more condensed molecular packing and also implying that these compounds could form morphologically stable films under Joule heating.^70–73^ The thermal morphological stability of DDPB, DBDPA and CDDPI thin film was examined by atomic force microscopy (AFM) measurement at room temperature and also at 90 °C for 12 h. The root-mean-square roughness (RMS) of DDPB (0.29 nm), DBDPA (0.21 nm) and CDDPI (0.34 nm) show that there is no substantial changes before and after annealing (90 °C, 12 h) which also supports the suitability of these emissive materials for fabrication of OLEDs^74–76^ (Fig. 3). The carrier injection at interface between different layers in OLEDs is essential for high performance devices, thus, it is important for the emissive layer to have shallow HOMO energy (*E*_HOMO_) for improving the hole injection.^77^ The electronic energies (HOMO and LUMO: Fig. 1) of the non-doped blue emitters such as DDPB, DBDPA and CDDPI have been examined from redox potentials measured from potential *vs.* current plot (Fig. 3). From the oxidative onset potential, the *E*_HOMO_ of −5.25 (DDPB), −5.14 eV (DBDPA) and −5.10 eV (CDDPI) (*E*_HOMO_ = *E*_ox_ + 4.8 eV) can be calculated and *E*_LUMO_ −2.39 (DDPB), −2.56 eV (DBDPA) and −2.59 eV (CDDPI) have been deduced from *E*_LUMO_ = *E*_HOMO_ − 1239/*λ*_onset_.^78^ The fully twisting molecular configuration of DDPB is likely to be the reason for higher energy gap of 2.86 eV (−2.58 eV – DBDPA; −2.51 eV – CDDPI). A single bond difference in the geometry of DDPB from DBDPA and CDDPI could change their photophysical properties and frontier energies. The space charge separation is found in these molecules which would be benefit for the injection of carrier from electrode.^79^ The partial overlap of HOMO/LUMO on phenanthrimidazole ring supports the charge transfer from π-linked binaphthyl to phenanthrimidazole moiety and the long π-linkers are beneficial to enhance the quantum yield. The DDPB, DBDPA and CDDPI ground state energy and their electron density delocalization at twist angle of 50° and 180° over HOMO and LUMO are shown in Fig. 4. Based on DFT energy, *ϕ* (50°) conformation of DDPB, DBDPA and CDDPI is more stable than *ϕ* (180°) conformation; in HOMO, the electron density is located on TPA and Cz and in LOMO the electron density is localized on imidazole moiety *i.e.*, the HOMO and LUMO orbitals are separated from each other, therefore, CT transition of 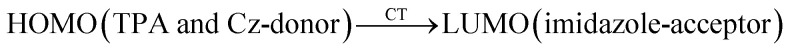 is possible. However, at 50° (low energy twisting conformation) the electron density is partially localized on the frontier orbitals of DDPB, DBDPA and CDDPI. Therefore at 50°, 
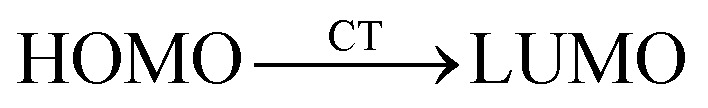
 transition is not pure CT but it is intercrossed between CT and LE transitions.^59^ The HOMO and LUMO of DDPB, DBDPA and CDDPI display adequate separation features and the differences are quite small which benefits the hole- and electron-transport properties (bipolar properties) and reduces the singlet–triplet splitting (Δ*E*_ST_).^80^ Hence, the HOMO and LUMO moieties individually undertake the electron and hole transport functions. The calculated electron/hole transfer integrals of CDDPI (0.23/0.31 eV), DDPB (0.24/0.39 eV) and DBDPA (0.26/0.48 eV) reveal that these materials are bipolar materials. Moreover, these compounds displays both reduction and oxidation behaviour, revealing that these compounds possess good electron and hole transport abilities, hence, the synthesized materials are bipolar transport materials.^81^

**Table 1:** Photophysical and thermal properties and device performances of DDPB, DBDPA and CDDPI

Parameters	DDPB	DBDPA	CDDPI
**Photophysical and thermal**	**Properties**		
a *λ*_ab_ (nm) (sol/film)	253, 371/254, 373	254, 379/258, 380	249, 365/252, 369
a *λ*_em_ (nm) (sol/film)	435/449	442/461	429/446
b *T*_m_/*T*_g_/*T*_d5_ (°C)	358/151/458	396/184/490	412/197/495
c *ϕ* (sol/film)	0.68/0.61	0.75/0.74	0.82/0.73
*τ* (ns)	5.9	6.1	5.1
d *E*_HOMO_/*E*_LUMO_ (eV)	−5.25/−2.39	−5.14/−2.56	−5.10/−2.59
*k*_r_ × 10^8^ (s^−1^)	1.1	1.2	1.6
*k*_nr_ × 10^8^ (s^−1^)	0.5	0.4	0.3

e *E*_g_ (eV)	−2.86	−2.58	−2.51
**Device efficiency**			
g *η*_IQE_ (%)	10.05	14.25	15.05
h *η*_s_ (%)	16.48	19.26	26.62
*V*_on_ (V)	4.3	3.7	3.0
*L* (cd m^−2^)	2010	3015	3992
f *η*_ex_ (%)	2.01	2.85	3.01
*η*_c_ (cd A^−1^)	1.61	2.01	2.56
*η*_p_ (lm W^−1^)	1.43	1.92	2.12
EL (nm)	447	459	444
CIE (*x*, *y*)	(0.16, 0.07)	(0.15, 0.12)	(0.15, 0.11)

a Normalized absorption (*λ*_ab_) and emission (*λ*_em_) spectra of DDPB, DBDPA and CDDPI in CH_2_Cl_2_ (10^−5^ M)/film.

b *T*_g_/*T*_d5_ – glass transition temperature/thermal decomposition temperature at a weight percentage of 95%.

c *ϕ* (soln/film) – PL quantum yield was calculated in dichloromethane/solid state quantum yield has been measured on the quartz plate using an integrating sphere.

d *E*_HOMO/LUMO_ – (*E*_HOMO_ = *E*_ox_ + 4.8 eV)/*E*_LUMO_ = *E*_HOMO_ − 1239/*λ*_onset_.

e *E*_g_ – energy gap (*E*_HOMO_–*E*_LUMO_).

f *η*_ex_ – external quantum efficiency; maximum internal quantum efficiency.

g *η*_IQE_ = *η*_ex_/*η*_out_, *η*_out_ light out coupling efficiency (−20%); excitation utilization efficiency.

h *η*_s_ = *η*_IQE_/*η*_PL_.

### 3.3. Photophysical properties and HLCT character

**Fig. 5:**
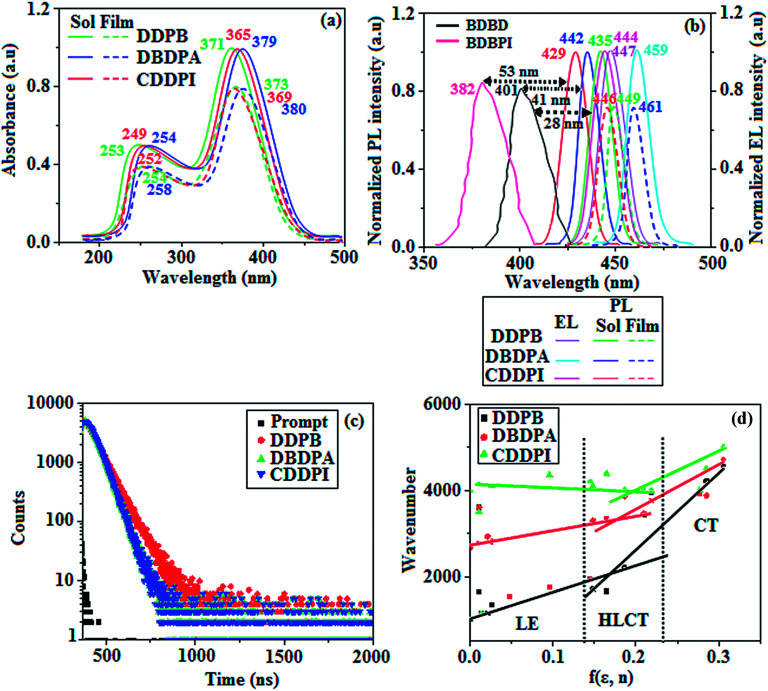
(a) Normalized optical absorption spectra of DDPB, DBDPA and CDDPI; (b) normalized emission spectra BDBD and BDBPI in CH_2_Cl_2_ (10^−5^ M) along with EL spectra of DDPB, DBDPA and CDDPI and (c) life time decay curve of DDPB, DBDPA and CDDPI (d) Lippert–Mataga plot of DDPB, DBDPA and CDDPI.

**Fig. 6:**
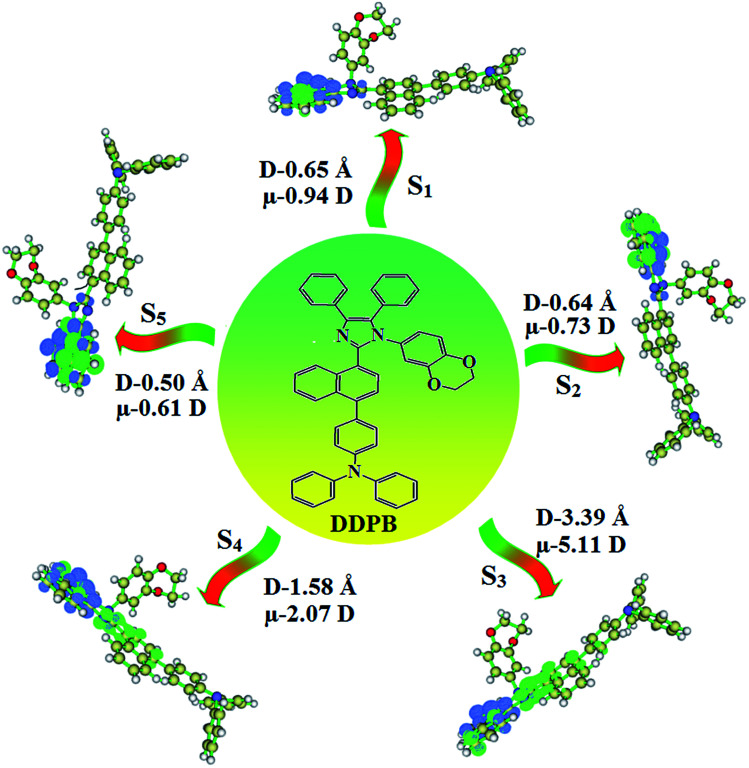
Hole and particle distribution of DDPB [S_1_–S_5_ states: 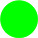-green increasing electron density and 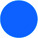-blue decreasing electron density [density = transition = *n*IOp(6/8 = 3)]].

**Fig. 7:**
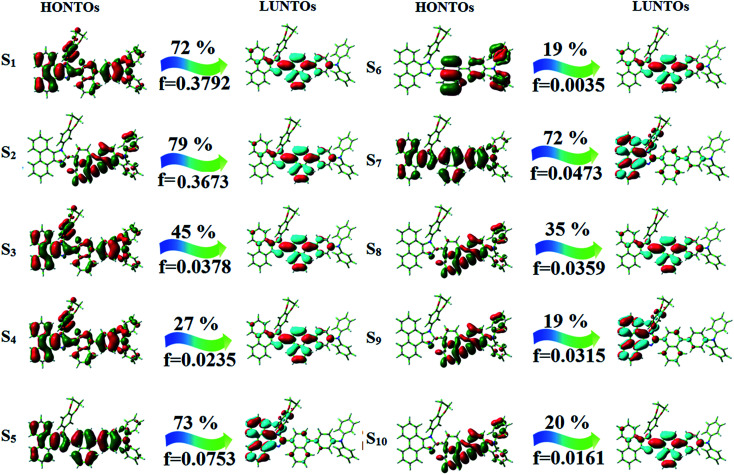
Highest occupied and lowest unoccupied natural transition orbitals of DBDPA.

The photophysical properties of DDPB, DBDPA and CDDPI were investigated in solution and solid by absorption (*λ*_abs_) and emission (*λ*_emi_) studies (Fig. 5, Table 1).^82^ The strong absorption around 250 nm is due to π–π* transition originates from phenyl to imidazole ring. In addition, the absorption around 370 nm is attributed to the intramolecular charge transfer from donor to acceptor unit.^83^ The D–π–A derivatives (DDPB, DBDPA and CDDPI) exhibit higher blue shift with higher molar absorptivity when compared to their parent compounds and this might be due to the presence of strong and weak electron donor triphenylamine and a carbazole moiety which is expected to enhance the efficiencies.^49^ The extinction coefficient of D–π–A molecules is higher due to increase of conjugation length.^84^ The DDPB, DBDPA and CDDPI show very strong *λ*_obs_ (absorption) at 371 nm (*ε*_max_ = 26 954 cm^−1^ M^−1^), 379 nm (*ε*_max_ = 26 385 cm^−1^ M^−1^) and 365 nm (*ε*_max_ = 27 397 cm^−1^ M^−1^) on comparison with parent compounds BDBD and BDBPI due to intramolecular CT transition from donor to acceptor (Fig. 5). The film state of blue emissive materials show absorption at 373 nm (DDPB), 380 nm (DBDPA) and 369 nm (CDDPI) and the small shift shows the existence of weak π–π* intermolecular stacking.^85^ The parent compounds of DDPB and DBDPA/CDDPI exhibit emission at 382 and 401 nm with vibronic nature. The DDPB and DBDPA show red shifted emission at 435 and 442/446 nm, respectively without vibronic nature (Fig. 5). The observed solvatochromic red shift with increase of solvent polarity 45 nm (DDPB), 32 nm (DBDPA) and 24 nm (CDDPI) confirmed that the low-lying excited CT state of DDPB, DBDPA and CDDPI possesses large dipole moment (Fig. 6, S9 and S10†)^51,86^ and the red shifted emission (Tables S1–S3†) could be attributed to the twisted conformation which enable for the easier charge transfer from donor to accept or *via* naphthyl linker. The intramolecular charge transfer is further confirmed by molecular electrostatic potential (MEP) (Fig. 1). Compared with solution, the small red shift in their corresponding film reveal that suppressed π–π* stacking exist in solid state.^87^ The emission of DDPB, DBDPA and CDDPI is observed at 449, 461 and 446 nm in solid and the full-width at half-maximum (FWHM) is around 30 nm: the red-shift progress cannot be due to aggregation in its solid state and might be from the change of excited state configuration.^9^ The phenanthroimidazole derivatives DDPB, DBDPA and CDDPI show blue emission at 435, 442 and 429 nm, respectively in CH_2_Cl_2_ (Fig. 5). The PL spectra gradually widened and their peaks show red shift with increase of solvent polarity which indicates that their excited state have strong CT character when compared to ground state and further stabilized by polar solvents.^87–89^ The calculated singlet energy/triplet energy (*E*_S_/*E*_T_) of DDPB (2.52/2.29 eV), DBDPA (2.60/2.25 eV) and CDDPI (2.58/2.23 eV) shows that they have high triplet energy to sensitize phosphorescent dopants with *E*_T_ below 2.3 eV. Compared with DBDPA, CDDPI exhibit higher blue shift in absorption and emission attributed to poor electron donor ability of Cz relative to TPA. The increased LE composition with decrease of CT in S_1_ emissive HLCT state is likely to be the reason for this blue shift. The FWHM in the absorption spectrum of CDDPI (24 nm) is narrowed compared to that of DBDPA (32 nm) and CDDPI (30 nm). This observation informed that decreased CT component of CDDPI in S_1_ state which is in good agreement with NTO description for S_0_ → S_1_ transition. The emission peak of DBDPA and CDDPI gives blue-shift relative to their parent compounds which is in controversy to the general observation *i.e.*, extension of π-conjugation leads to red shifted emission.^89^ In addition to that there is an overlap between UV and PL spectra of both DBDPA and CDDPI because of enhanced LE character in DBDPA and CDDPI than their respective parent compounds. The CDDPI exhibits solvatochromic red shift (24 nm) which is smaller than DBDPA (32 nm) (Fig. S11, Tables S3 and S2†). Similarly, a small absorption shift about 28 nm and 20 nm has been observed for CDDPI and DBDPA, respectively (Fig. S11, Tables S3 and S2†). Solvatochromic shifts confirmed that low-lying S_1_ excited state of CDDPI and DBDPA must possess CT character.^90,91^ The % of CT character in S_1_ state of CDDPI is lower than DBDPA whereas % of LE character of CDDPI is higher than DBDPA (Table S4†). In S_0_–S_1_ and S_0_–S_2_ transitions, the HLCT was composed with CT state and LE state and exhibit larger oscillator strength [*f*_S_0_–S_1__ = 0.5191 (DDPB): 0.6814 (CDDPI): 0.3792 (DBDPA) and *f*_S_0_–S_2__ = 0.3023 (DDPB): 0.6712 (CDDPI): 0.3673 (DBDPA)] compared with other S_0_–S_*n*_ (*n* = 3, 4…) transitions as a result of major LE character in the HLCT state (Table 2; Fig. 7 – DBDPA; Fig. S12† – DDPB; Fig. S13† – CDDPI) which is necessary for higher efficiency OLEDs.^92^ In DBDPA and CDDPI, the hole and particle of S_0_–S_1_ and S_0_–S_2_ transitions of NTOs contained two transition configurations with close contributions; one squint towards the LE state and the other was like HLCT state (Fig. 4). These results implied that DBDPA and CDDPI exhibits a better mixed LE and CT state and the LE state dominated the fluorescence and intersection angle appeared between two directions in the phenanthrimidazole ring, namely one along C2-substituted direction and another along N1-substituted direction. Most of the CT component was nearly perpendicular to that of LE in the same mixed transition configuration which was a special excited state of the D–π–A structure. This novel mixed state led to low oscillator strength in DBDPA, (*f*_S_0_–S_1__ – 0.3792) than that of CDDPI (*f*_S_0_–S_1__ – 0.6814) and the transition energy barrier was very little from S_1_ to S_4_ (0.04 eV). Such slight differences usually cause stronger orbital coupling leading to free exciton transformation between the four excited states at room temperature and the emission species change or the number increases with changes in the external environment such as thermal activation. This internal conversion between different excited states would cause non-radiative decay ratio to increase photoluminescence efficiency with larger oscillator strength which is confirmed by the optical measurements.

**Table 2:** Calculated energies (*E*) and oscillator strength (*f*) of S_1_–S_10_ transitions from NTO of DDPB, DBDPA and CDDPI

Transitions	DDPB			DBDPA			CDDPI		
	*E*	*f*	NTO	*E*	*f*	NTO	*E*	*f*	NTO
S_0_ → S_1_	1.32	0.5191	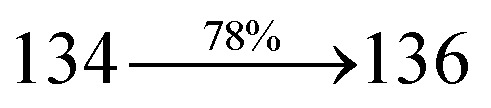	2.75	0.3792	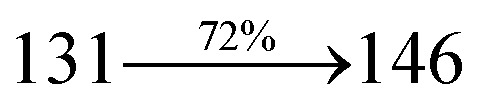	1.85	0.6814	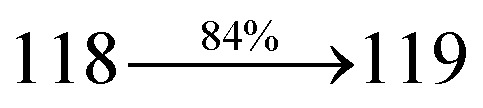
S_0_ → S_2_	2.02	0.3023	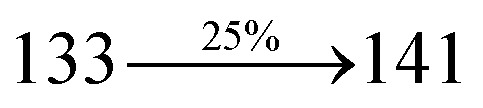	3.05	0.3673	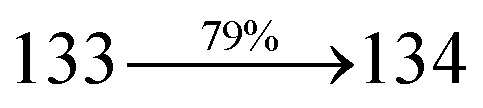	1.94	0.6712	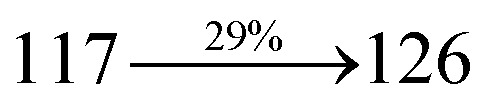
			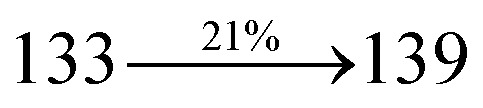						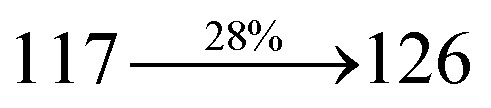
S_0_ → S_3_	2.40	0.2983	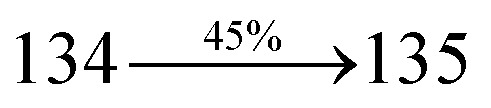	3.44	0.0378	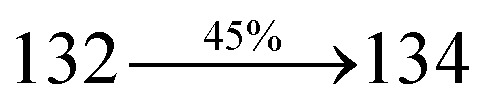	2.15	0.0485	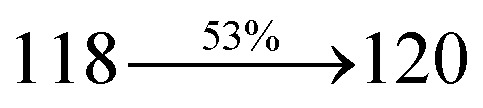
S_0_ → S_4_	2.58	0.2428	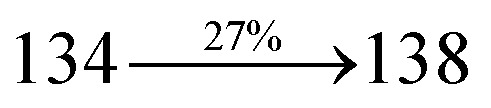	3.61	0.0235	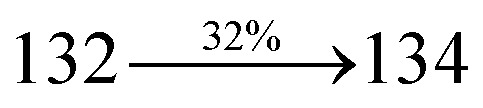	2.36	0.0620	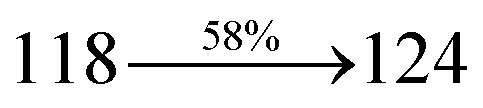
S_0_ → S_5_	2.79	0.0292	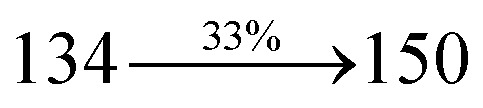	3.70	0.0753	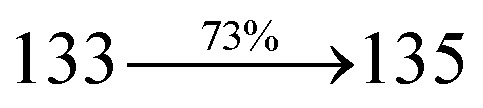	2.86	0.0414	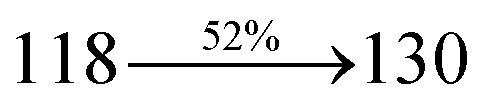
S_0_ → S_6_	2.94	0.0678	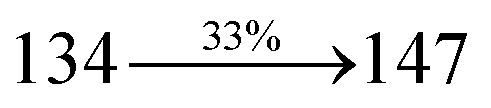	3.73	0.0035	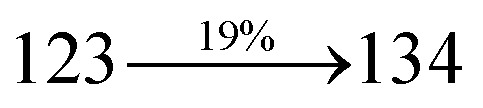	2.96	0.0188	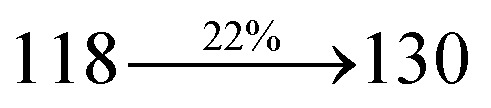
			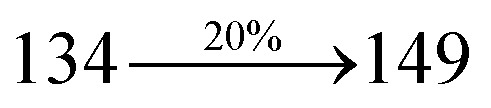						
			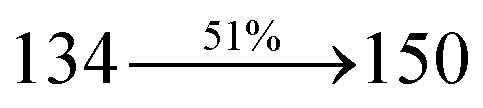						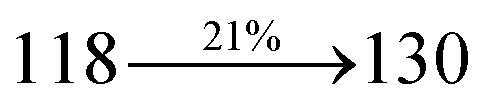 1
			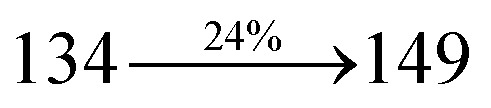						
S_0_ → S_7_	3.22	0.0489	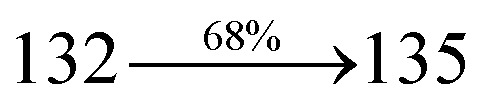	3.77	0.0473	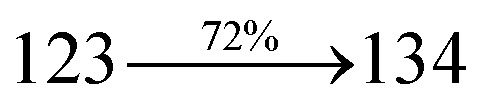	3.15	0.0553	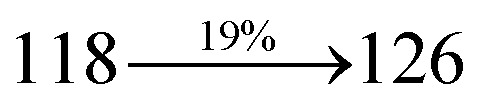
S_0_ → S_8_	3.52	0.1293	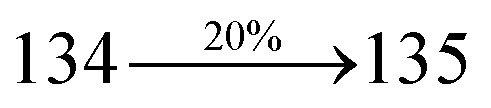	3.80	0.0359	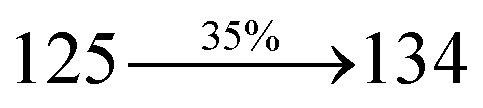	3.25	0.3998	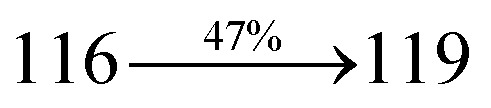
S_0_ → S_9_	3.57	0.0142	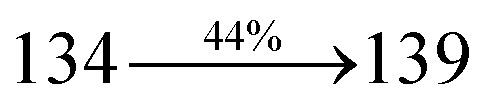	3.83	0.0315	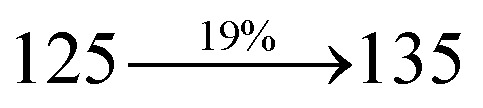	3.45	0.1324	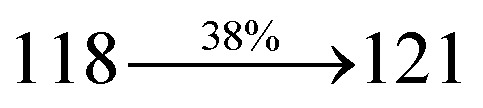
S_0_ → S_10_	3.61	0.0271	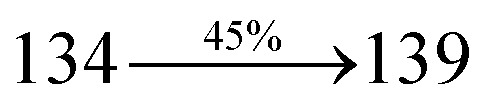	3.86	0.0161	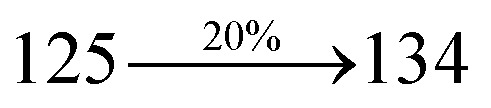	3.50	0.0119	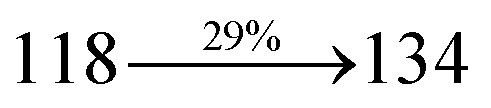

The new born blue emitters show high quantum yield (solution/film) of DDPB (0.68/0.61), DBDPA (0.75/0.74) and CDDPI (0.82/0.73) and the high fluorescence yield is essential for efficient blue OLEDs (Table 1). The improved quantum yield is attributed to decreased proportion of non-radiative transition because of molecular interactions.^78^ Incorporation of binaphthyl into bulky phenanthrimidazole ring enhanced the intermolecular steric hindrance forced the molecule to form a more twisted structure when packing in solid which results in less aggregation and lower quantum yield in their solid state. The lower yield of DDPB is due to increased excited state intra molecular vibration results from the fully twisted DDPB whereas the high rigid geometry of DBDPA and CDDPI effectively reduced the radiative exciton which results high yield. The *k*_r_/*k*_nr_ (radiative transition rate and non-radiative transition rate) have been calculated from lifetime (*τ*) and quantum yield (*ϕ*) (Table 1). To analyze the relative contribution of radiative and non-radiative relaxation processes in the excited state deactivation, the radiative (*k*_r_) and non-radiative (*k*_nr_) decay constants were calculated: *k*_r_ = *τ*/*Φ*: 1.1 s^−1^ (DDPB); 1.2 s^−1^(DBDPA) and 1.6 s^−1^ (CDDPI); *k*_nr_ = *τ*/(1 − *Φ*): 0.5 s^−1^ (DDPB); 0.4 s^−1^ (DBDPA) and 0.3 s^−1^ (CDDPI) (Fig. 5). The CDDPI shows larger radiative rate constant (*k*_r_) and smaller non-radiative rate constant (*k*_nr_) than those of DDPB and DBDPA.

### 3.4. Solvatochromism for HLCT character

The solvatochromic effect using Lippert–Mataga plot has been displayed in Fig. 5 (Tables S1–S3†). When solvent polarity increased the blue emitters exhibit a larger red shift which supports the charge transfer (CT) in these molecules.^92^ From Lippert–Mataga plot, the ground state dipole moment (*μ*_g_) can be calculated: *hc*(*υ̃*_abs_ − *υ̃*_flu_) = *hc*(*hcυ̃*^νac^_abs_ − *hcυ̃*^νac^_flu_) + 2(*μ*_e_ − *μ*_g_)^2^/*a*_o_^3^[(*ε* − 1/2*ε* + 1) − 1/2(*n*^2^ − 1/2*n*^2^ + 1)] [*μ*_g_ and *μ*_e_ – ground state and excited state dipolemoment, *υ̃*_abs_ and *υ̃*^νac^_abs_ – solvent-equilibrated absorption maxima (*λ*_abs_) and extrapolated to gas phase, *υ̃*_flu_ and *υ̃*^νac^_flu_ – solvent equilibrated fluorescence maxima (*λ*_emi_) and extrapolated to gas-phase, respectively, *a*_o_ – onsager cavity and *ε* and *n* solvent dielectric constant and refractive index, respectively]. The non-linear correlation of Stokes shift *vs.* solvent polarity function reveal that there is transformation of fitted line between ethyl ether and methylene chloride: non-linear correlation supports the presence of both locally excited state (LE) and charge transfer excited state (CT). The ground state dipole (*μ*_g_) of blue emitting materials, DDPB, DBDPA and CDDPI could be estimated from density functional theory (DFT) calculation as, 3.12, 5.02 and 7.1 D, respectively which is attributed by local exciton (LE) transition and *μ*_e_ in high polar solvents is 21.9, 23.4 and 23.9 D, respectively.^92^ The large *μ*_e_ in high polar medium is in close with *μ*_e_ of charge-transfer molecule, 4-(*N*,*N*-dimethylamino)benzonitrile (23.0 D).^93^ All these results show that CT dominates in more polar medium and LE dominates in low polar solvent and there is mixed contribution of LE and CT in medium polar solvents. The high oscillator strength of S_1_ state of CDDPI results in higher PL efficiency (*η*_PL_). Molecular modification from TPA to Cz causes an increasing % LE in S_1_ emissive state and enhanced *η*_PL_ of CDDPI. The overlap density between hole and particle depend upon the configuration of donor–acceptor architecture and the magnitude of overlap intensity tuned the % LE and % CT in S_1_ state (Fig. 6 – DDPB, Fig. S9† – DBDPA, Fig. S10† – CDDPI). The two different excited states of DDPB, DBDPA and CDDPI confirmed the two independent slopes of non-linear fitted line,^91^*i.e.*, LE and CT intercrossed excited state: in high polarity solvents CT state dominates, in low polar solvents LE state dominates and in moderate polar solvents intercrossed excited state of LE and CT exist. The intercrossed coupling of LE with CT states generates new hybridized local and charge transfer state (HLCT). The HLCT state in moderate polar solvent was confirmed by mono exponential time (Fig. 5) which supports the D–π–A molecular design.^75,94^ The LE and CT states of emissive materials DDPB, DBDPA and CDDPI show non-uniform properties in different polar solvents because of different excited-state dipole moments. As the polarity increases, CT state is stabilized due to strong interaction of the solvent field with CT excited state (large dipole moment) and LE remains unchanged,^95^ however, in low-polarity solvents, the LE being stabilized (low-lying excited state). The *λ*_emi_ of DBDPA and CDDPI film is close with that in ether which confirmed HLCT state formation.^91^

**Scheme 2:**
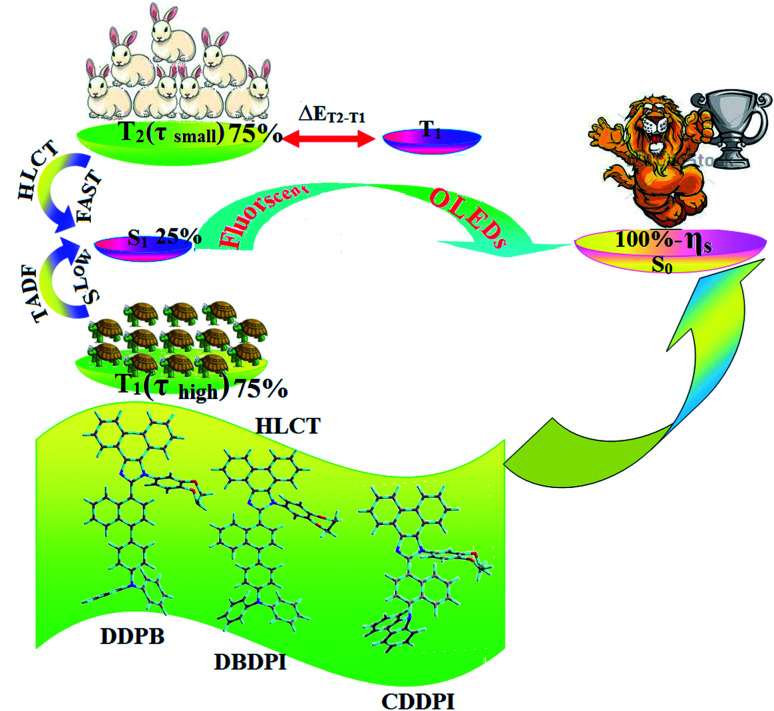
Effect of TADF and HLCT on 100% exciton utilization efficiency (*η*_s_).

The excited-state properties of DBDPA (Fig. 7), DDPB (Fig. S12†) and CDDPI (Fig. S13†) were analyzed using natural transition orbitals (NTOs).^41^ The electron density delocalization over hole and particles confirmed that in S_0_ → S_1_ transition, LE state dominates with minor contribution of CT state in HLCT and shows higher when compared with S_0_ → S_2_ or other S_0_ → S_10_ transitions [*f*_S_0_→S_1__ = 0.5191 > *f*_S_0_→S_2__ = 0.3023 (DDPB): *f*_S_0_→S_1__ = 0.3792 > *f*_S_0_→S_2__ = 0.3673 (DBDPA): *f*_S_0_→S_1__ = 0.6814 > *f*_S_0_→S_2__ = 0.6712 (CDDPI)]. This is further evidence for higher % LE in HLCT state and is highly need for efficient OLED performances. The lower oscillator strength of DDPB reveal that DDPB exhibit completely hybridized LE and CT states than DBDPA and CDDPI and the eigen value (>0.95) further supports the better mixed LE and CT excited state to about 78% of transition which is supported by Δ*r* > 2 for S_1_–S_10_ of DDPB, DBDPA and CDDPI (Tables S1–S3†). The electron density distribution on hole and particle of DBDPA and CDDPI were entirely differing from DDPB. The excitation energies of LE and CT states of the title materials were used as a tool to confirm the formation of HLCT state^56^ (Scheme 2, Tables 3–5).

**Table 3:** Excitation energy (*E*, eV), excitation co-efficient (*ε*), overlap integral (Δ*r*, Å) for singlet and triplet states of DDPB

State	Singlet			Triplet		
	*E*	*ε*	Δ*r*	*E*	*ε*	Δ*r*
1	1.3069	0.4716	2.2387	0.4016	0.4711	2.0157
2	2.0159	0.4033	2.6236	1.3068	0.4232	2.2177
3	2.4046	0.4522	4.8614	1.0737	0.4387	1.9531
4	1.7468	0.4260	4.9963	1.4491	0.4256	3.1194
5	2.7853	0.4327	3.4986	1.8658	0.4193	3.9187
6	2.9430	0.4250	3.0147	2.3521	0.3010	2.3141
7	1.7501	0.4195	1.4300	2.5457	0.4334	1.5606
8	3.5184	0.3508	4.5728	2.7755	0.3649	3.4988
9	3.5663	0.4113	4.2429	2.8379	0.2631	3.0845
10	3.6105	0.3381	4.4619	2.9442	0.1891	2.9672

**Table 4:** Excitation energy (*E*, eV), excitation co-efficient (*ε*), overlap integral (Δ*r*, Å) for singlet and triplet states of DBDPA

State	Singlet			Triplet		
	*E*	*ε*	Δ*r*	*E*	*ε*	Δ*r*
1	1.7539	0.4535	1.8665	1.0999	0.3407	1.2953
2	3.0502	0.4372	0.8836	1.7538	0.2743	3.5474
3	3.4377	0.3555	1.8897	2.6921	0.2809	2.7663
4	3.6106	0.3722	3.0340	2.7554	0.2250	3.1037
5	3.6978	0.3272	2.6956	2.8864	0.2590	2.5155
6	2.3895	0.3825	0.7581	2.0085	0.2620	3.2297
7	3.4778	0.3346	3.8811	2.2316	0.2572	1.8697
8	3.7976	0.2677	2.8953	2.2368	0.4446	1.8799
9	3.8287	0.2937	3.1646	2.4858	0.2958	3.0048
10	2.3105	0.3122	3.6380	2.5909	0.2057	1.4642

**Table 5:** Excitation energy (*E*, eV), excitation co-efficient (*ε*), overlap integral (Δ*r*, Å) for singlet and triplet states of CDDPI

State	Singlet			Triplet		
	*E*	*ε*	Δ*r*	*E*	*ε*	Δ*r*
1	1.4084	0.4348	2.0371	0.2898	0.4348	1.0780
2	1.9364	0.4457	5.6501	1.5683	0.4110	2.4346
3	1.4235	0.4520	4.9302	1.5863	0.9194	2.5722
4	2.3572	0.4627	2.0641	1.6340	0.6205	1.9457
5	2.8613	0.4260	1.9569	1.6904	0.6353	1.9177
6	2.9554	0.4091	3.9229	1.8919	0.7509	1.8641
7	3.1549	0.4237	2.4004	1.9710	0.6499	3.1570
8	3.2532	0.4152	3.9584	2.0172	0.6481	2.9625
9	3.4267	0.4419	4.9631	2.3568	0.8882	2.6737
10	3.4993	0.4282	2.6747	2.4278	0.6423	2.8688

**Fig. 8:**
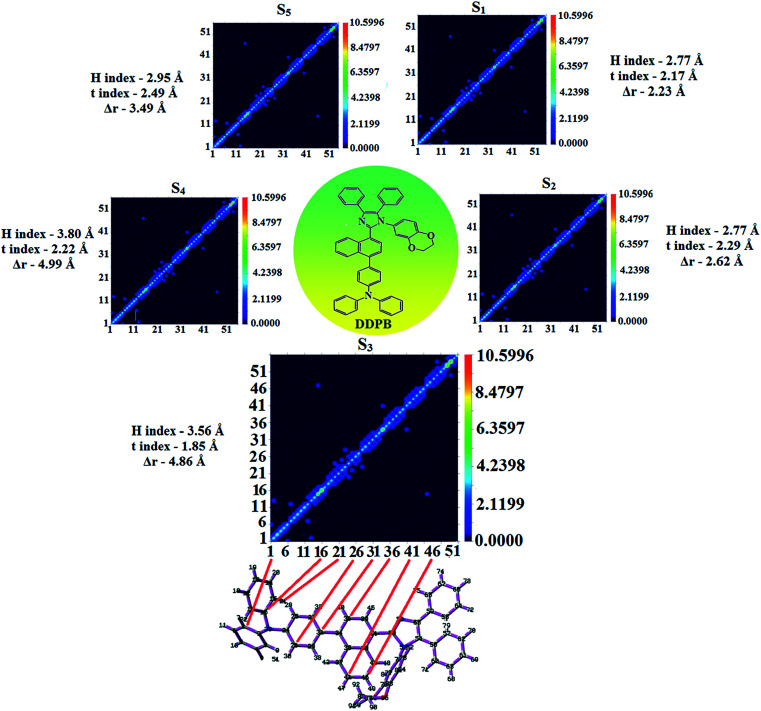
Computed contour plots of transition density matrices (TDM) of DDPB [density = transition = *n*/IOp(6/8 = 3)].

**Fig. 9:**
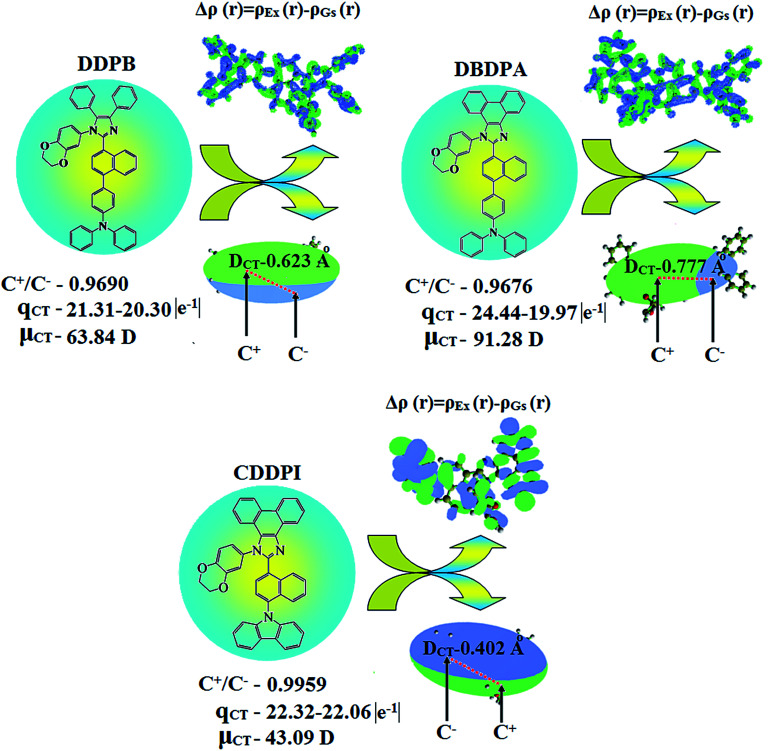
Differences in total density for ground and excited states [Δ*ρ*(*r*) = *ρ*_Ex_(*r*) − *ρ*_Gs_(*r*); isosurface for DDPB, DBDPA, CDDPI, (0.0000006 a.u.) and for DDPB (0.15 a.u.)]; graphical representation of *D*_CT_ and centroids of charges [*C*+(*r*)/*C*−(*r*); isosurface for DBDPA (0.29 a.u.) and for CDDPI (0.1 a.u.)].

In DDPB, CT state is stabilized than LE state and the energy gap is too small results effective hybridization whereas in DBDPA and CDDPI, the LE state is stabilized than CT state due to enhanced π-conjugation results weaker interstate coupling. The overlap between hole and particle of DDPB, DBDPA and CDDPI is displayed in Fig. 6, S9 and S10,† respectively. The more similar hole–electron wave function indicates the efficient hybridization between LE and CT states. The composition of HLCT in DDPB (Fig. 8), DBDPA (Fig. S14†) and CDDPI (Fig. S15†) can be analyzed by transition density matrix (TDM). The diagonal part reflects the LE component localized on main backbone while off-diagonal region represents CT component. Analysis of integral of electron, integral of hole and their overlap, integral of transition density and distance between hole and electron are displayed in Table S4† (DDPB), Table S5† (DBDPA) and Table S6† (CDDPI) and these excited state parameters reveal that the integral of hole and electron of DDPB is less than DBDPA and CDDPI whereas the electron density is close to the ideal value of zero which indicates the medium quality grid is enough for visualization. Computed electron–hole properties, distance between hole and electron, transition density, *H* and *t* indexes and RMSD of electron and hole of DDPB, DBDPA and CDDPI are displayed in Tables S7–S9,† respectively. Transition dipole moment calculated by integrating the same at uniform grid is almost same for both cases. The integral overlap of hole–electron distribution is a measure of spatial separation of hole and electron which is close to zero for DBDPA and CDDPI. Distance between centroid of hole and electron is a measure of CT length: the larger CT value indicates charge transfer takes place with longer length (Fig. 9, Table 6). The excited state characteristic of DDPB, DBDPA and CDDPI further supports that these materials capable of transporting electrons and holes in the fabricated devices. The coexisting LE/CT composition in DDPB, DBDPA and CDDPI harvested high *η*_PL_ and high *η*_s_ and enhanced OLEDs efficiencies. The new born blue emitters show high quantum yield (*s*/*f*) of DDPB (0.68/0.61), DBDPA (0.75/0.74) and CDDPI (0.82/0.73) and high fluorescence efficiencies are essential for efficient OLEDs (Table 1). The improved quantum yield is attributed to decreased proportion of the non-radiative transition because of molecular interactions, such as intermolecular interaction between cyano group and phenanthrimidazole plane. Incorporation of binaphthyl into bulky phenanthrimidazole enhanced the intermolecular steric hindrance forced the molecule to form a more twisted structure when packing in solid which results in less aggregation and lower quantum yield in solid state. It is a rare model for which the PL spectra are unchanged and the yields are increased by inserting bulky naphthyl group.

**Table 6:** Transferred charges (*q*_CT_), barycentres of electron density loss (*R*_−_)/gain (*R*_+_), distance between two barycenters (*D*_CT_), dipole moment of CT (*μ*_CT_), RMSD of +ve/−ve parts, CT indices (*H* & *t*) and overlap integral of *C*+/*C*− of DDPB, DBDPA and CDDPI

Blue emissive & host materials	*q*_CT_ |*e*^−1^|	*R*_+_ (Å)			*R*_−_ (Å)			*D*_CT_ (Å)	*μ*_CT_ (D)	+ve RMSD	−ve RMSD	*H*/*t* indices (Å)	Overlap integral (*C*+/*C*−)
		*x*	*y*	*z*	*x*	*y*	*z*						
DDPB	21.319–20.306	−0.03	−0.86	0.80	−0.12	−1.37	−0.25	0.623	63.84	11.87	12.53	6.45/6.19	0.9690
DBDPA	24.448–19.979	−2.35	1.05	−0.37	−2.93	−0.27	−0.13	0.777	91.28	11.05	12.93	6.32/5.75	0.9676
CDDPI	22.321–22.062	−1.89	0.99	0.28	−2.11	1.57	0.73	0.402	43.09	11.97	11.75	6.27/5.98	0.9959

### 3.5. Quasi-equivalent hybridization

**Fig. 10:**
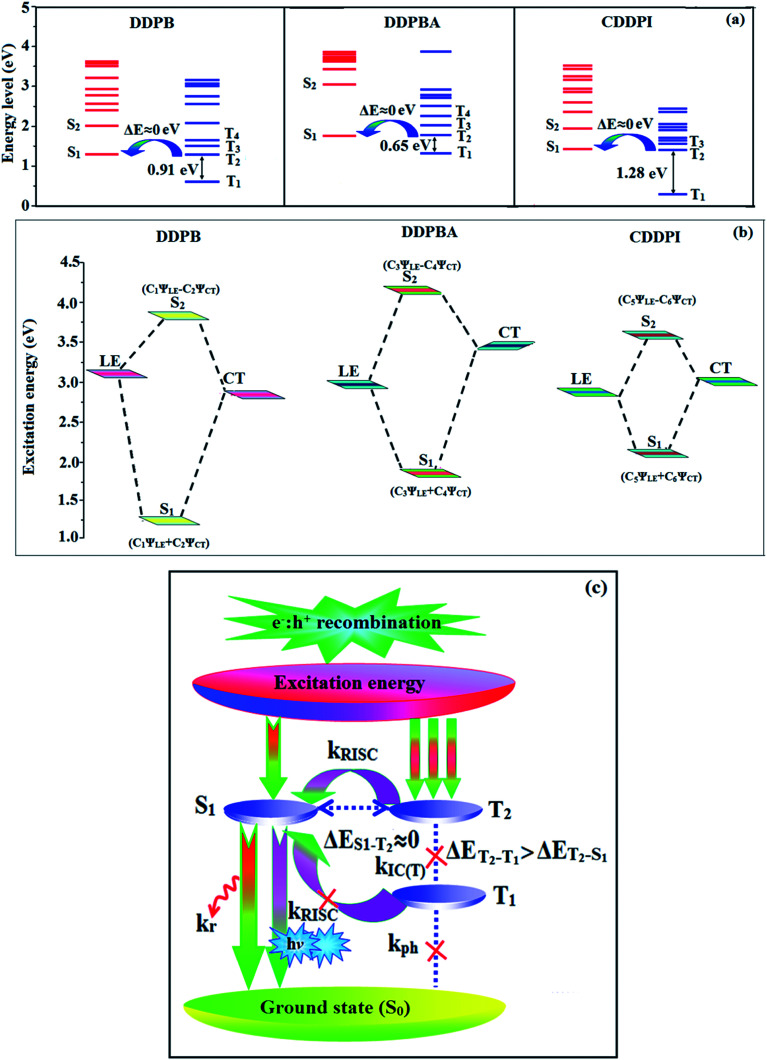
(a) Schematic diagram of hybridization processes of LE and CT states of DDPB, DBDPA, CDDPI; (b) energy level of singlet (S) and triplet (T) states of DDPB, DBDPA, CDDPI; (c) scheme of exciton decay process after hole and electron recombination in OLEDs of D–π–A molecules.

The HONTOs and LUNTOs of S_1_ and S_2_ excited states of DDPB, DBDPA and CDDPI exhibit a hybrid splitting state character that derives from interstate coupling of LE and CT levels to form HLCT (Tables 3–5). The hole contour on Cz or TPA moiety are in the opposite phase between S_1_ and S_2_ states whereas the particle on phenanthrimidazole moiety is same between S_1_ and S_2_ states for DDPB, DBDPA and CDDPI, respectively. This implied that the interstate hybridization coupling occurs through the positive and negative linear combination between LE and CT state wave function: *Ψ*_S_1_/S_2__ = *c*_LE_*Ψ*_LE_ ± *c*_CT_*Ψ*_CT_. The percentage of CT level of CDDPI (62%) is less than that of DDPB (80%) and DBDPA (90%) as a result of weak donor ability of Cz than TPA results LE dominated S_1_ state in CDDPI (LE ∼ 45%), DDPB (LE ∼ 15%) and DBDPA (LE ∼ 10%) (Table S10†). As a result, CDDPI should exhibit higher photoluminescence efficiency (*η*_PL_) and blue shifted emission relative to DDPB and DBDPA. The S_1_ and S_2_ excited states of DDPB, DBDPA and CDDPI are similar in energy, oscillator strength and HONTOs and LUNTOs distribution which indicate a quasi-equivalent hybridization between LE and CT states due to their almost iso-energetic initial states. In contrast, for nonequivalent hybridization the S_1_ and S_2_ excited states have quite significant difference in energy, oscillator strength and NTO image which is caused by non-equivalent hybridization between LE and CT initial states. Compared with non-equivalent hybridization, the quasi-equivalent hybridization is expected to achieve high *η*_PL_ and high *η*_S_. The more balanced LE and CT components in HLCT state of DDPB, DBDPA and CDDPI enhanced the EL efficiency. The formation of HLCT state can be analyzed through the excitation energies of LE and CT states (Tables 3–5). A large energy gap between T_1_ and T_2_ for CDDPI (1.28 eV), DDPB (0.91 eV) and DBDPA (0.65 eV) arising from the same phenanthrimidazole acceptor and the energy gap between T_1_ and T_2_ of CDDPI is larger than DDPB and DBDPA (Fig. 10).^96,97^ A very small Δ*E*_ST_ ≈ 0 is observed between S_1_ and T_3_ states facilitating RISC (T_2_ → S_1_) process as a result of their HLCT state character. The increased LE component in S_1_ state of CDDPI enhanced the photoluminescence efficiency (*η*_PL_), and high exciton utilization efficiency (*η*_S_) and external quantum efficiency (*η*_ex_) have been harvested when compared with TPA emitters (Table 1). Excited state characters play a key role in PL and electroluminescence (EL) properties of OLEDs. Charge-transfer state is beneficial to enhance the singlet exciton utilization in fluorescent OLEDs by RISC due to small singlet and triplet energy splitting (Δ*E*_ST_) in CT exciton. However, the dominant CT component in the emissive state reduces the PL efficiency in such materials. Here, the strategy is to carry out for fine excited state modulation to achieve combination of high PL efficiency using locally emissive (LE) component and high exciton utilizing CT component in one excited state. As a result, a quasi-equivalent hybridization of LE and CT components obtained in the emissive state upon addition of binaphthyl bridge in the newly synthesized material. Similar hole–electron wave function between S_1_ and S_2_ is observed in DDPB, DBDPA and CDDPI indicates a quasi-equivalent hybridization between LE and CT states as a result of their almost isoenergies of initial LE and CT states. Therefore, degree of hybridization between LE and CT states depends not only initial *E*_LE_–*E*_CT_ energy gap but also their interstate coupling strength.^98^ Compared with non-equivalent hybridization, quasi-equivalent hybridization is expected to achieve the combination of high *η*_PL_ and high *η*_S_ to maximize EL efficiency of fluorescent OLED materials due to more balanced LE and CT components in HLCT state of DDPB, DBDPA and CDDPI. In DDPB, DBDPA and CDDPI, the LE state is stabilized than CT state and energy gap (*E*_S_2__–*E*_S_1__) is small when compared with their parent compounds results quasi hybridization. In the case of CDDPI, the energy gap (*E*_S_2__–*E*_S_1__) is reduced more when compared with DBDPA results effective hybridization and improves OLED efficiency. The qualitatively calculated percentage of LE and CT in S_1_–S_10_ and T_1_–T_10_ states are displayed in Table S10.† This also supports that HLCT state also contributes to hybridization apart from LE and CT states.

### 3.6. Single carrier devices

**Fig. 11:**
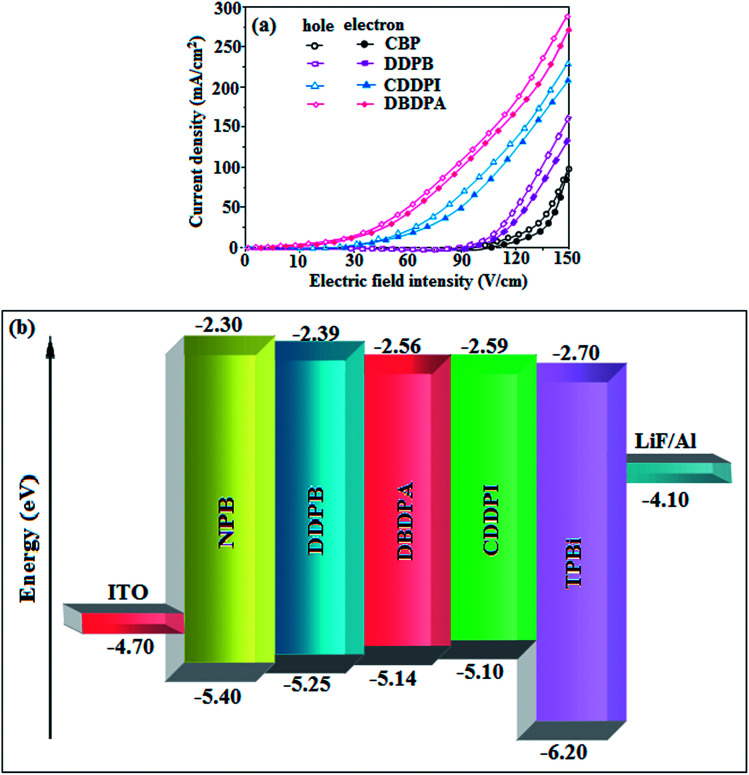
(a) Hole-only and electron-only devices based on DDPB, DBDPA and CDDPI; (b) energy level diagram of non-doped devices.

To evaluate their carrier injection and transport properties, hole-only and electron-only devices have been fabricated: (a) ITO/HATCN (8 nm)/DDPB/DBDPA/CDDPI (50 nm)/HATCN (8 nm)/LiF (1 nm)/Al (100 nm) (hole-only device IV) and (b) ITO/TPBi (8 nm)/DDPB/DBDPA/CDDPI (50 nm)/TPBi (8 nm)/LiF (1 nm)/Al (100 nm) (electron-only device V). Fig. 11 shows the current density *versus* voltage characteristics of hole-only and electron-only devices. The electron current density of DDPB, DBDPA and CDDPI based device is higher than CBP-based device which reveal that these materials have effective electron injection and transport properties than CBP. The difference in current density between hole-only and electron-only devices based on DDPB, DBDPA and CDDPI is much smaller than that based on CBP at same voltage suggesting these materials are potential bipolar material capable of transporting electrons and holes in devices.^99–101^

### 3.7. Electroluminescent studies

The effective film forming properties of emissive materials are important for device efficiency. The better nanoscale morphology of annealed DBDPA thin film is attributed to low turn-on voltage. The only difference between DDPB and DBDPA and CDDPI is one single bond which affects the OLED performances. Energy-level diagram of the materials used for the fabrication of devices are shown in Fig. 11. The TADF material will show flat decay curve due to the time consuming TADF process for the exciton conversion from triplet to singlet however, the observed single-exponential sharp decay of DDPB, DBDPA and CDDPI shows that the radiative exciton are short-lived component without TADF contribution (Fig. 5). The lifetime measurement reveal that this intercrossed excited state in different polar solvents should be a hybridized local and charge transfer state (HLCT) instead of two species state through a simple addition of LE and CT. The mono exponential demonstrates that the intercrossed LE and CT in the moderate polar solvent formed as one hybridized HLCT state which supports the molecular design (Scheme 2). The exciton utilization efficiency (*η*_S_) in DDPB, DBDPA and CDDPI are of neither TTA nor TADF mechanism.

**Fig. 12:**
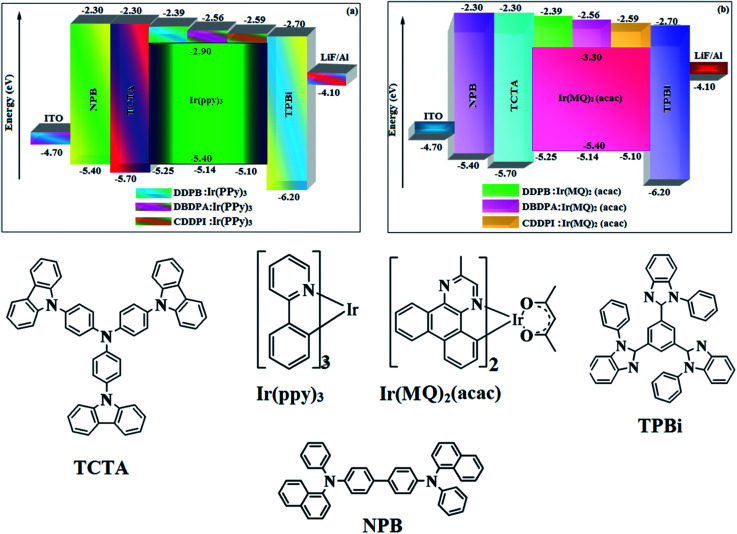
Energy level diagram of green (a) and red (b) devices with molecular structures of functional materials used in devices.

**Fig. 13:**
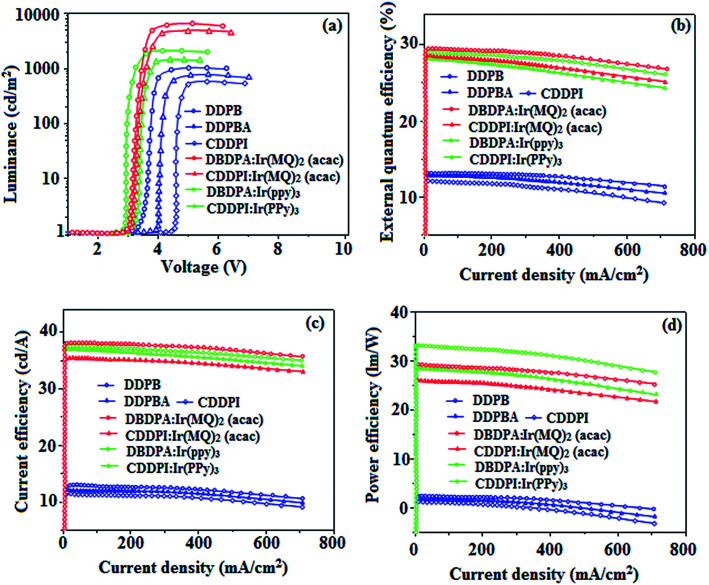
Device performances of DDPB, DBDPA, CDDPI, DBDPA:Ir(ppy)_3_, CDDPI:Ir(PPy)_3_, DBDPA:Ir(MQ)_2_(acac) and CDDPI:Ir(MQ)_2_(acac): (a) luminance–voltage; (b) external quantum efficiency–current density; (c) current efficiency–current density and (d) power efficiency–current density.

The blue device with the configuration of ITO/NPB (60 nm)/DDPB/DBDPA/CDDPI (30 nm)/LiF (1 nm)/Al (100 nm) have been fabricated (Fig. 12). The electroluminescence (EL) spectra of the devices are similar to their PL spectra which shows both EL and PL originates from the same radiative decay of the singlet exciton. High device performances at low-turn on voltage are extracted from non-doped devices based on DDPB, DBDPA and CDDPI (Table 1). The CDDPI based device shows high efficiencies (*η*_c_ – 2.56 cd A^−1^; *η*_p_ – 2.12 lm W^−1^; *η*_ex_ – 3.01%: Fig. 13) at 3.0 V with CIE (0.15, 0.11) and EL is 444 nm (FWHM-45 nm) than DBDPA (*η*_c_ – 2.01 cd A^−1^; *η*_p_ – 1.92 lm W^−1^; *η*_ex_ – 2.85%) at 3.7 V with CIE (0.15, 0.13) and EL is 459 nm (FWHM-75 nm). The high *η*_ex_ harvested from CDDPI and DBDPA based device is due to the co-emission from intercrossed excited state of LE and CT; the isoenergies of singlet (^1^CT) and triplet (^3^CT) make ^3^CT → ^1^CT transition as spin-allowed transition.^102^ The device with DDPB exhibit deep blue EL emission at 447 nm (narrow FWHM-37 nm) with CIE (0.16, 0.07) and the efficiencies are (*η*_c_ – 1.61 cd A^−1^; *η*_p_ – 1.43 lm W^−1^; *η*_ex_ – 2.01%) at 4.3 V. The EL difference between DDPB and DBDPA is ∼12 nm whereas DBDPA exhibit blue emission with 75 nm normal FWHM. The EL and PL spectra of DDPB, DBDPA and CDDPI at 77 K and room temperature are shown in Fig. 5. The FWHM of PL spectrum of DDPB was gradually narrower (48 nm) from room temperature [77 K (40 nm)] to EL spectra (37 nm) whereas for DBDPA and CDDPI, the FWHM of EL spectra (75 nm – DBDPA and 69 nm – CDDPI) was larger than that of PL spectra at room temperature (65 nm – DBDPA and 60 nm – CDDPI) [77 K: (56 nm – DBDPA) and 50 nm – CDDPI]. On comparison with PL emission of DBDPA and CDDPI, the narrow EL spectrum is explained by weak microcavity effect and suppressed intramolecular vibration.^103^ The inevitable vibration splitting in the strongly rigid phenanthro[9,10-*d*]-imidazole structure of DBDPA and CDDPI may be enhanced in OLEDs to show large full peak width with red-shifted CIE. The external quantum efficiency of OLEDs can be calculated as follows: EQE = *η*_out_ × *η*_rc_ × *η*_*γ*_ × *Φ*_PL_,^104^ [*η*_out_ – light-out-coupling efficiency (20%), *η*_rc_ – product of the charge recombination efficiency (100%), *η*_*γ*_ – efficiency of radiative exciton production (25%) and *Φ*_PL_ – photoluminescence quantum yield of the emitters]. The *η*_r_ calculated for DDPB (13–16%), DBDPA (19–21%) and CDDPI (16–21%) indicates *γ* is less than 100% due to very small unbalanced carrier transportation.^105^ This result could be attributed more balanced charge-transporting properties within the emissive layer achieved by better charge injection provided by hole transport layer. The *η*_IQE_ can be calculated from *η*_EQE_/*η*_out_ as DDPB (10.1%), DBDPA (14.3%) and CDDPI (15.1%) and maximum *η*_s_ of DDPB (16.5%), DBDPA (19.3%) and CDDPI (26.6%) of EL devices can be estimated using the equation *η*_s_ = *η*_res_ × *η*_PL_ × *η*_out_/*η*_EL_, where *η*_out_ (≈1/2*n*^2^) is light out coupling efficiency (≈20%); *η*_rec_ is efficiency for electron hole recombination (100%). The enhanced *η*_s_ and *η*_IQE_ is probably due to the maintained CT component of D–π–A compounds. The device efficiencies are compared with already reported non-doped emitters efficiencies^58,106–114^ (Table 7) which shows that the newly synthesized non-doped devices based on DDPB, DBDPA and CDDPI are among the best in terms of efficiencies. These experimental results demonstrated that the additional triplet exciton have been utilized in the OLED applications for the HLCT character of DDPB, DBDPA and CDDPI as shown in Scheme 2 and showing the accuracy for our molecular design strategy. Devices with DDPB, DBDPA and CDDPI show maximum luminance (*L*) of 2010, 3015 and 3992 cd m^−2^, respectively. The EL brightness has a linear relationship with current density for these compounds indicating that the contribution from triplet–triplet annihilation was insignificant.^115^ The emission wavelength of DDPB, DBDPA and CDDPI in film is close to that in ethyl ether which supports the HLCT state formed in DDPB, DBDPA and CDDPI film.

**Table 7:** Summary of device efficiencies with reported non-doped emitters

Emitter	*V*_on_ (V)	*L* (cd m^−2^)	EL (nm)	*η*_c_ (cd A^−1^)	*η*_p_ (lm W^−1^)	CIE (*x*, *y*)	Ref.
**DDPB**	**4.3**	**2010**	**447**	**1.61**	**1.43**	**(0.16, 0.07)**	**This work**
**DBDPA**	**3.7**	**3015**	**459**	**2.01**	**1.92**	**(0.15, 0.12)**	**This work**
**CDDPI**	**3.0**	**3992**	**444**	**2.56**	**2.12**	**(0.15, 0.11)**	**This work**
PPI	3.8	3307	412	0.71	0.40	(0.161, 0.065)	106
mTPA-PPI	3.2	4065	404	0.84	0.48	(0.161, 0.049)	106
L-BPPI (50 nm)	8.5	70	440	0.01	—	(0.16, 0.11)	107
L-BPPI (40 nm)	6.5	295	440	0.13	—	(0.16, 0.11)	107
L-BPPI (30 nm)	5.0	420	440	0.40	—	(0.16, 0.10)	107
L-BPPI (20 nm)	4.5	391	440	0.68	—	(0.16, 0.10)	107
Z-BPPI (50 nm)	6.5	105	440	0.07	—	(0.17, 0.12)	107
Z-BPPI (40 nm)	5.0	502	440	0.34	—	(0.16, 0.12)	107
Z-BPPI (30 nm)	4.5	267	440	0.45	—	(0.16, 0.12)	107
Z-BPPI (20 nm)	5.0	100	440	0.31	—	(0.16, 0.11)	107
TPA-PIM	—	4510	420	1.14	0.79	(0.161, 0.046)	58*b*
MADN (BUBD)	7.8	—	440	2.1	—	(0.15, 0.10)	108
CPPPI	—	3322	420	0.65	0.48	(0.165, 0.050)	109
PPICPPPI	—	4329	428	1.53	0.86	(0.166, 0.056)	109
PhBPI	2.8	—	450	1.87	1.85	—	110
Bilayer-TPBI	3.2	—	468	2.03	1.00	(0.15, 0.15)	111
TPA-BPI	2.8	—	448	1.83	1.58	(0.15, 0.09)	112
DPVBi	7.5	—	457	0.03	—	(0.15, 0.13)	113
DPVICz	4.2	—	470	0.92	—	(0.15, 0.22)	113
DPVTCz	3.8	—	470	1.94	—	(0.14, 0.22)	113
3,6-DPVTCz	5.0	—	449	0.11	—	(0.15, 0.11)	113
PEDOt-PSS: 3 (100 nm)	4.0	2800	460	0.61	0.14	(0.15, 0.14)	114
PEDOt-PSS: 3 (50 nm)	3	10 600	407	1.68	1.10	(0.16, 0.13)	114
PEDOt-PSS: 4 (40 nm)	2.5	21 200	392	1.90	1.55	(0.16, 0.14)	114

The calculated triplet energy (*E*_S_/*E*_T_) of DDPB (2.29 eV), DBDPA (2.25 eV) and CDDPI (2.23 eV) shows that they have high triplet energy to sensitize phosphorescent dopants with *E*_T_ below 2.3 eV. These DBDPA and CDDPI are also employed as host materials for green and red phosphorescent dopants. The fabricated green and red devices are having the configuration of ITO/NPB (40 nm)/TCTA (5 nm)/DBDPA (30 nm): 5 wt% Ir(ppy)_3_/CDDPI (30 nm): 5 wt% Ir(ppy)_3_/TPBI (50 nm)/LiF (1 nm)/Al (100 nm): ITO/NPB (40 nm)/TCTA (5 nm)/DBDPA (30 nm): 8 wt% Ir(MQ)_2_(acac)/CDDPI (30 nm): 8 wt% Ir(MQ)_2_(acac)/TPBI (50 nm)/LiF (1 nm)/Al (100 nm), respectively (Fig. 12), [Ir(ppy)_3_-*fac*-tris(2-phenylpyridine) iridium(iii) and Ir(MQ)_2_(acac)-bis(2-methyldibenzo-[*f*,*h*]quinoxaline) acetylacetonate iridium(iii) are used as emissive layers for green and red devices, respectively]. The device performances are shown in Fig. 13. The EL spectra are similar to PL spectra of the doped thin films (Fig. 5). The green device (432 nm) with CDDPI (30 nm): 5 wt% Ir(ppy)_3_ exhibits maximum luminance of 8812 cd m^−2^, maximum current and power efficiencies are of 27.5 cd A^−1^ and 33.0 lm W^−1^, respectively at 2.7 V with CIE (0.31, 0.60) (Table 4). The maximum external quantum efficiencies of the devices based on CDDPI:Ir(ppy)_3_ and DBDPA:Ir(ppy)_3_ [*η*_c_ – 27.5 cd A^−1^; *η*_p_ – 33.0 lm W^−1^; *L* – 8609 cd m^−2^; CIE (0.31, 0.60) with CIE (0.64, 0.34)] are 19.0 and 18.2%, respectively. Similar to green devices, red device (640 nm) with CDDPI:Ir(MQ)_2_(acac) exhibits maximum luminance of 39 461 cd m^−2^ and excellent EL efficiencies (*η*_ex_ – 19.2%; *η*_c_ – 27.9 cd A^−1^; *η*_p_ – 29.2 lm W^−1^ with CIE (0.64, 0.34) on compared with DBDPA:Ir(MQ)_2_(acac) based on device (*L* – 37 621 cd m^−2^; *η*_ex_ – 18.5%; *η*_c_ – 25.2 cd A^−1^; *η*_p_ – 25.8 lm W^−1^; CIE (0.64, 0.34) with EL 641 nm. The above experimental results demonstrate that CDDPI and DBDPA are universal host materials for green and red phosphorescent emitters (Table 8). The device performances reveal that CDDPI and DBDPA are universal host materials for green and red phosphorescent emitters.

**Table 8:** Electroluminescent efficiencies of green DBDPA:Ir(ppy)_3_, CDDPI:Ir(PPy)_3_ and red DBDPA:Ir(MQ)_2_(acac), CDDPI:Ir(MQ)_2_(acac)

Emitters	*V*_1000_ (V)	*L* (cd m^−2^)	*η*_ex_ (%)	*η*_c_ (cd A^−1^)	*η*_p_ (lm W^−1^)	CIE (*x*, *y*)	EL (nm)
DBDPA:Ir(ppy)_3_	3.0	8609	18.2	27.0	28.6	0.31, 0.60	436
CDDPI:Ir(PPy)_3_	2.7	8812	19.0	27.5	33.0	0.31, 0.60	432
DBDPA:Ir(MQ)_2_(acac)	3.2	37 621	18.5	25.2	25.8	0.64, 0.34	641
CDDPI:Ir(MQ)_2_(acac)	3.0	39 461	19.2	27.9	29.2	0.64, 0.34	640

## 4. Conclusion

In conclusion, the blue emissive, fully twisting DDPB based OLEDs exhibit quantum efficiency of 2.01% (FHWM of 37 nm) with CIE (0.16, 0.07). The *λ*_emi_ of DBDPA film is close with that in ether which confirmed HLCT state formation. The fully twisted DDPB with strong intramolecular vibration and weak microcavity effect produced narrow EL. The external quantum efficiency harvested from DBDPA based device is 2.85% with maximum current and power efficiency of 2.01 cd A^−1^ and 1.92 lm W^−1^, respectively. The enhanced *η*_s_ [DDPB – 16.5%, DBDPA – 19.3% and CDDPI – 26.7%] and *η*_IQE_ [DDPB – 10.1%, DBDPA – 14.3% and CDDPI – 15.1%] is probably due to the maintained CT component of D–π–A compounds. The blue emissive materials CDDPI and DBDPA used as a host to construct green and red phosphorescent OLEDs. The green device based on CDDPI (30 nm): 5 wt% Ir(ppy)_3_ exhibits maximum luminance of 8812 cd m^−2^, maximum current and power efficiencies are of 27.5 cd A^−1^ and 33.0 lm W^−1^, respectively at 2.7 V and red device based on CDDPI:Ir(MQ)_2_(acac) exhibits maximum luminance of 39 661 cd m^−2^ and excellent EL efficiencies (*η*_ex_ – 19.2%; *η*_c_ – 27.9 cd A^−1^; *η*_p_ – 29.2 lm W^−1^ with CIE (0.64, 0.34)) on compared with DBDPA:Ir(MQ)_2_(acac) based on device (*L* – 37 621 cd m^−2^; *η*_ex_ – 18.5%; *η*_c_ – 25.2 cd A^−1^; *η*_p_ – 25.8 lm W^−1^ with CIE (0.64, 0.34)). Fluorescent D–π–A emitters with HLCT state would be an effective practical strategy to develop low-cost and high-efficient blue organic electroluminescent materials.

## Conflicts of interest

There are no conflicts to declare.

## Supplementary Material

RA-009-C9RA00135B-s001
