# Supplementary material for: Efficient electroluminescent hybridized local and charge-transfer host materials with small singlet–triplet splitting to enhance exciton utilization efficiency: excited state transition configuration
Source: RSC Adv. 2019 Feb 26;9(12):6658–80. doi: 10.1039/c9ra00135b (PMC9060926; doi:10.1039/c9ra00135b)
Supplement: RA-009-C9RA00135B-s001 [file RA-009-C9RA00135B-s001.pdf]

Electronic Supplementary Material (ESI) for RSC Advances.
This journal is © The Royal Society of Chemistry 2019

Supporting information

Efficient electroluminescent hybridized local and charge-transfer host materials with small singlet–triplet splitting to enhance exciton utilization efficiency: Excited state transition configuration

Jayaraman Jayabharathi*, Venugopal Thanikachalam, Ganapathy Abirama Sundari
Department of Chemistry, Annamalai University, Annamalainagar-608002, Tamilnadu, India
* Tel: +91 9443940735; *E-mail address:jtchalam2005@yahoo.co.in*

* Address for correspondence

Dr. J. Jayabharathi
Professor of Chemistry
Department of Chemistry
Annamalai University
Annamalai nagar 608 002
Tamilnadu, India.
E-mail: jtchalam2005@yahoo.co.in

Contents

SI–I: Charge-Transfer indexes

SI–XV: Figures

SI–X: Tables

SI–I: Charge–Transfer indexes

The hole–particle pair interactions have been related to thedistance covered during the excitations one possibledescriptorΔr index could be used to calculate the average distance which is weighted in functionof the excitation coefficients.

$$\Delta r = \frac{\Sigma_{ia} k_{ia}^2 \left| \langle \varphi_a | r | \varphi_a \rangle - \langle \varphi_i | r | \varphi_i \rangle \right|}{\Sigma_{ia} K_{ia}^2}$$ …………….. (S1)

where$\left| \langle \varphi_i | r | \varphi_i \rangle \right|$ is the norm of the orbital centroid [1–4].Δr–index will be expressed in Å.

The density variation associated to the electronic transition is given by

$$\Delta\rho(r) = \rho_{EX}(r) - \rho_{GS}(r)$$ .... (S2)

where$\rho_{GS}(r)$and $\rho_{EX}(r)$are the electronic densities of to the ground and excited states, respectively. Two functions, $\rho_+(r)$ and $\rho_-(r)$, corresponds to the points in space where an increment or a depletion of the density upon absorption is produced and they can be defined as follows:

$$\rho_+(r) = \begin{Bmatrix} \Delta\rho(r) if \Delta\rho(r) > 0 \\ 0 \quad if \Delta\rho(r) < 0 \end{Bmatrix}$$ .... (S3)

$$\rho_-(r) = \begin{Bmatrix} \Delta\rho(r) if \Delta\rho(r) < 0 \\ 0 \quad if \Delta\rho(r) > 0 \end{Bmatrix}$$ .... (S4)

The barycenters of the spatial regions R_+ and R_- are related with $\rho_+(r)$ and $\rho_-(r)$ andare shown as

$$R_+ = \frac{\int r\rho_+(r)dr}{\int \rho_+(r)dr} = (x_+, y_+, z_+)$$ .... (S5)

$$R_- = \frac{\int r\rho_-(r)dr}{\int \rho_-(r)dr} = (x_-, y_-, z_-)$$ .... (S6)

The spatial distance (D_{CT}) between the two barycentersR$_+$ and R$_–$ of density distributionscan thus be used to measurethe CT excitation length

$$D_{CT} = |R_+ - R_-| \text{.................. (S7)}$$

The transferred charge (q_{CT}) can be obtained by integrating over all space $\rho_+(\rho_-)$,. Variation indipole moment between the ground and the excited states (μ_{CT}) can be computed by the following relation:

$$\|\mu_{CT}\| = D_{CT}\int \rho_+(r)dr = D_{CT}\int \rho_-(r)dr \text{......................... (S8)}$$

$$= D_{CT}q_{CT} \text{................. (S9)}$$

The difference between the dipole moments$\|\mu_{CT}\|$have been computed for the ground and the excited states $\Delta\mu_{ES\text{–}GS}$. The two centroids of charges ($C^+/C^–$) associated to the positive and negative density regions are calculated as follows. First the root–mean–square deviations along the three axis (σ_{aj}, j = x, y, z; a = + or –) are computed as

$$\sigma_{a,j} = \sqrt{\frac{\sum_i \rho_a(r_i)(j_i - j_a)^2}{\sum_i \rho_a(r_i)}} \text{........................... (S10)}$$

The two centroids (C_+ and $C_–$) are defined as

$$C_+(r) = A_+ e\left(-\frac{(x - x_+)^2}{2\sigma_{+x}^2} - \frac{(y - y_+)^2}{2\sigma_{+y}^2} - \frac{(z - z_+)^2}{2\sigma_{+z}^2}\right) \text{.................... (S11)}$$

$$C_{-+}(r) = A_- e\left(-\frac{(x - x_-)^2}{2\sigma_{-x}^2} - \frac{(y - y_-)^2}{2\sigma_{-y}^2} - \frac{(z - z_-)^2}{2\sigma_{-z}^2}\right) \text{.................... (S12)}$$

The normalization factors (A+ and A_) are used to impose the integrated charge on the centroid to be equal to the corresponding density change integrated in the whole space:

$$A_+ = \frac{\int \rho_+(r)dr}{\int e(-\frac{(x - x_-)^2}{2\sigma^2_{+x}} - \frac{(y - y_-)^2}{2\sigma^2_{+y}} - \frac{(z - z_-)^2}{2\sigma^2_{+z}})dr} \quad \text{.................. (S13)}$$

$$A_- = \frac{\int \rho_-(r)dr}{\int e(-\frac{(x - x_-)^2}{2\sigma^2_{-x}} - \frac{(y - y_-)^2}{2\sigma^2_{-y}} - \frac{(z - z_-)^2}{2\sigma^2_{-z}})dr} \quad \text{.................. (S14)}$$

H index is defined as half of the sum of the centroids axis along the D–A direction, if the D–A direction is along the X axis, H is defined by the relation:

$$H = \frac{\sigma_{+x} + \sigma_{-x}}{2} \quad \text{.................... (S15)}$$

The centroid along X axis is expected.The t intexrepresents the difference between D_{CT} and H:

$$t = D_{CT} - H \quad \text{............... (S16)}$$

SI-XV: Figures

Figure S1. H^1 NMR spectrum of DDPB

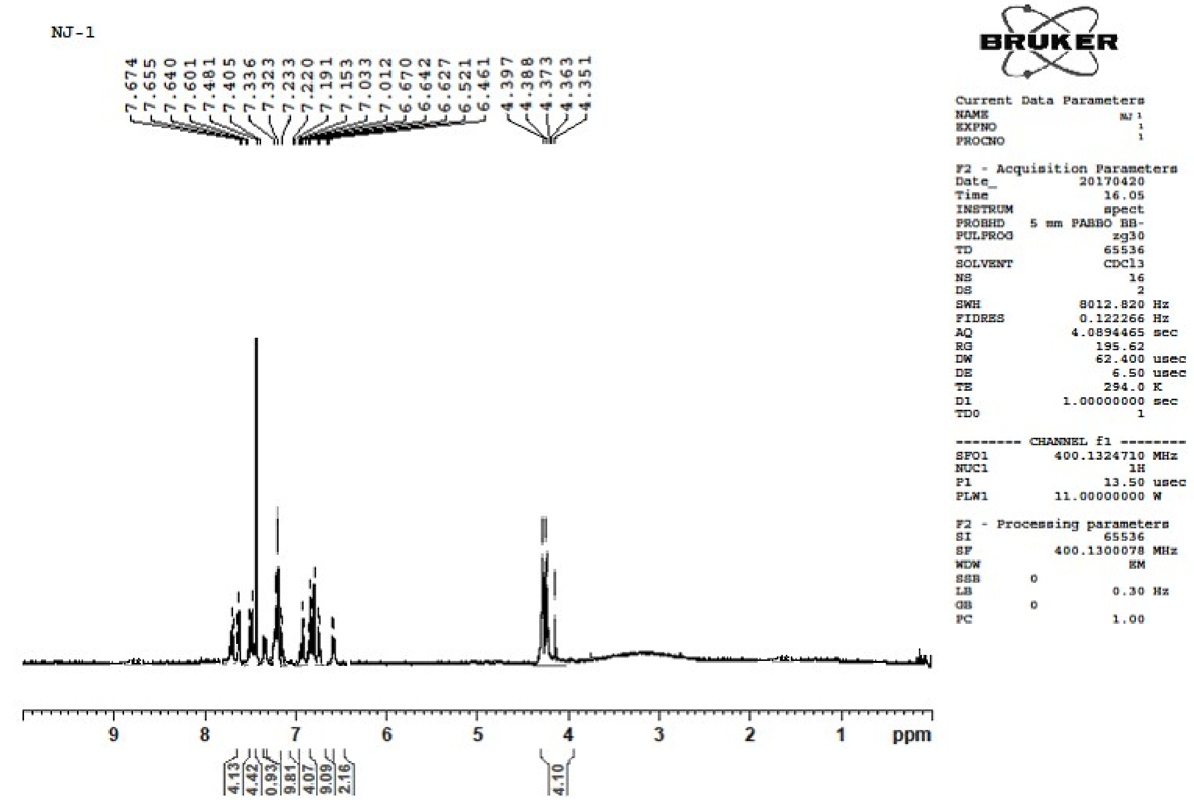

Figure S2. C^{13} NMR spectrum of DDPB

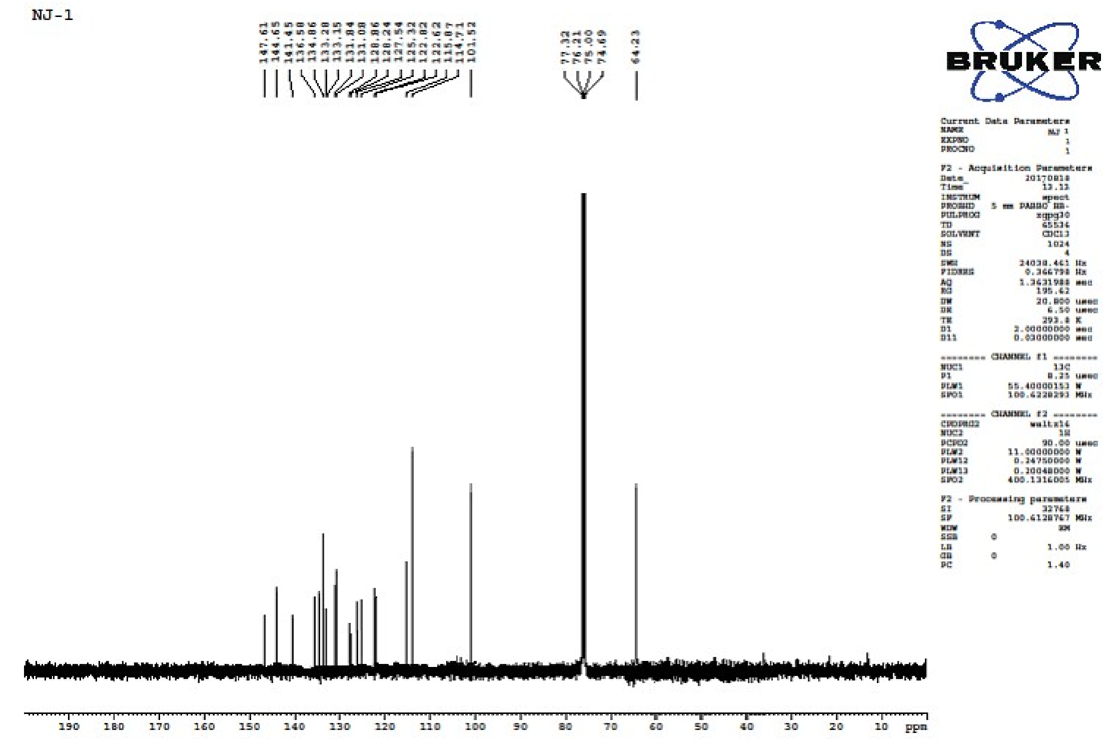

Figure S3. H^1 NMR spectrum of DBDPA

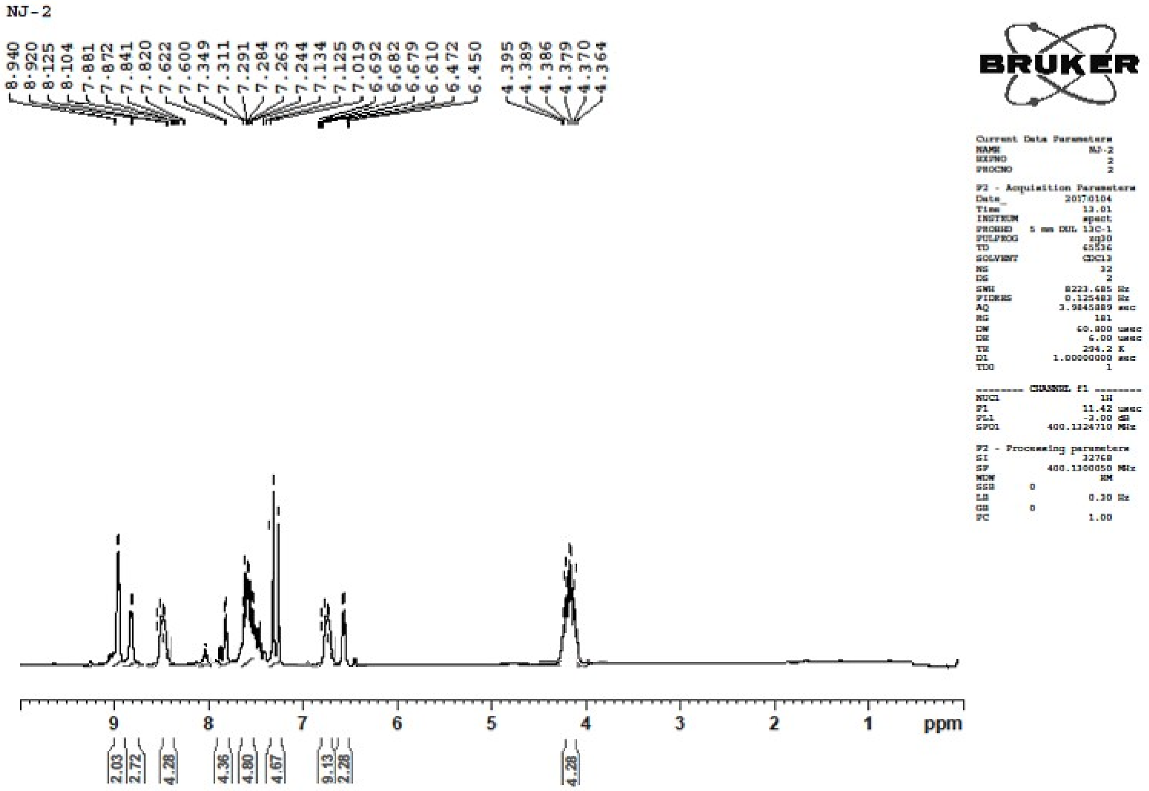

Figure S4. C^{13} NMR spectrum of DBDPA

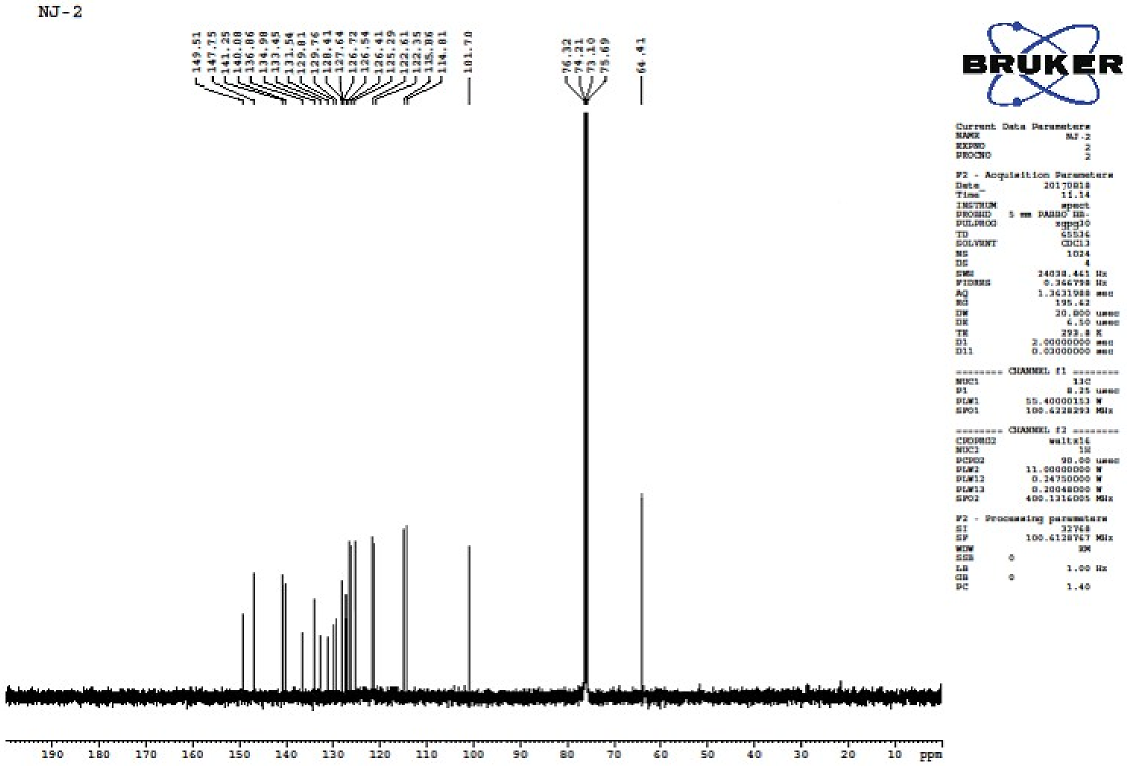

Figure S5. H^{1} NMR spectrum of CDDPI

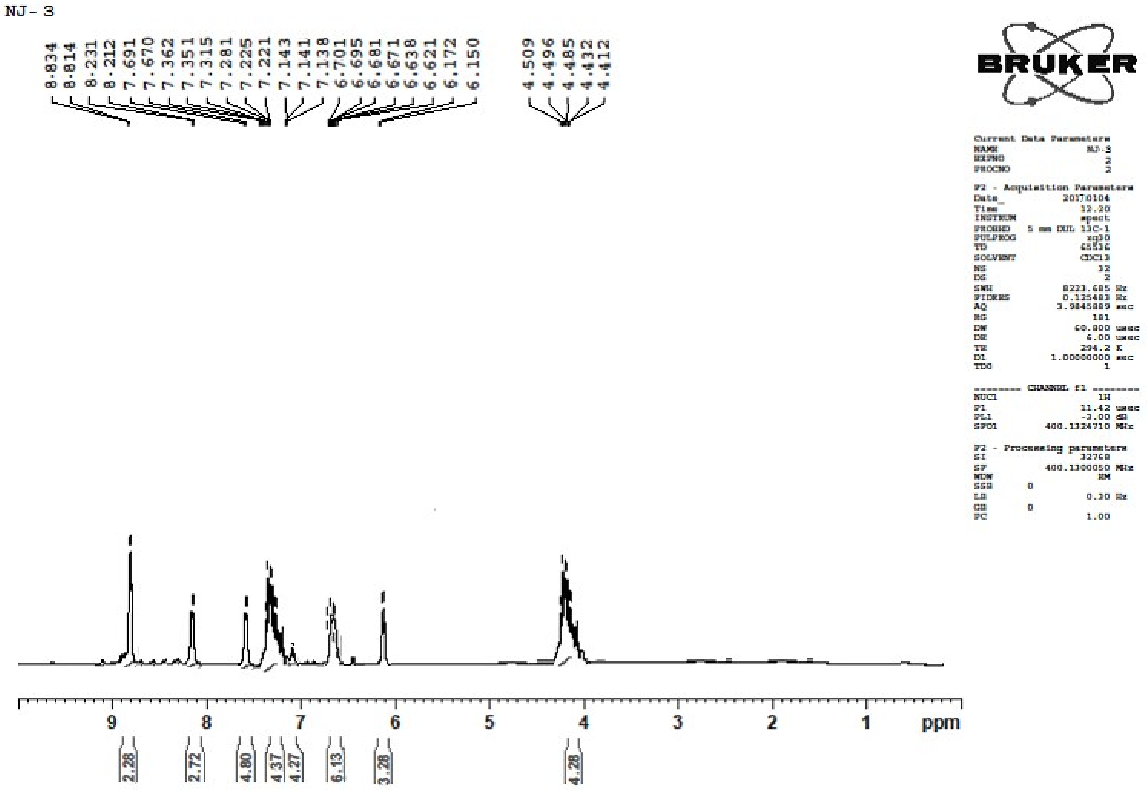

Figure S6. C^{13} NMR spectrum of CDDPI

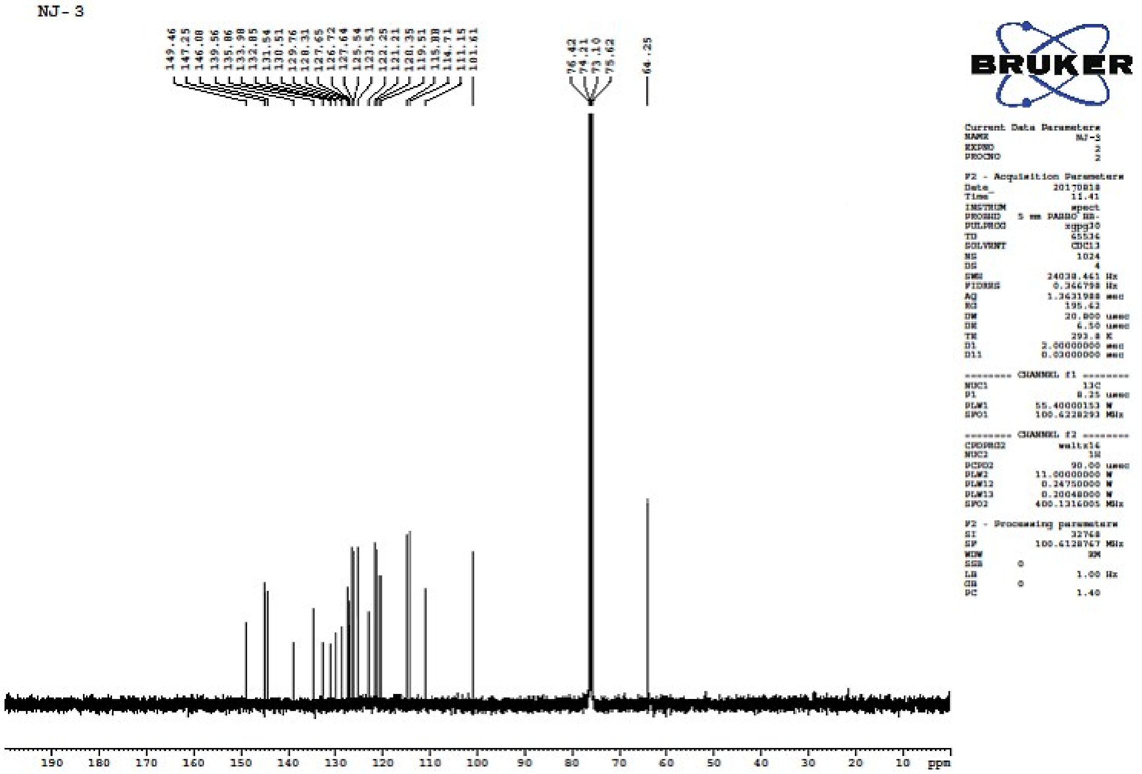

Figure S7. MALDI-TOF mass spectrum of (a) DDPB, (b) DBDPA and (c) CDDPI

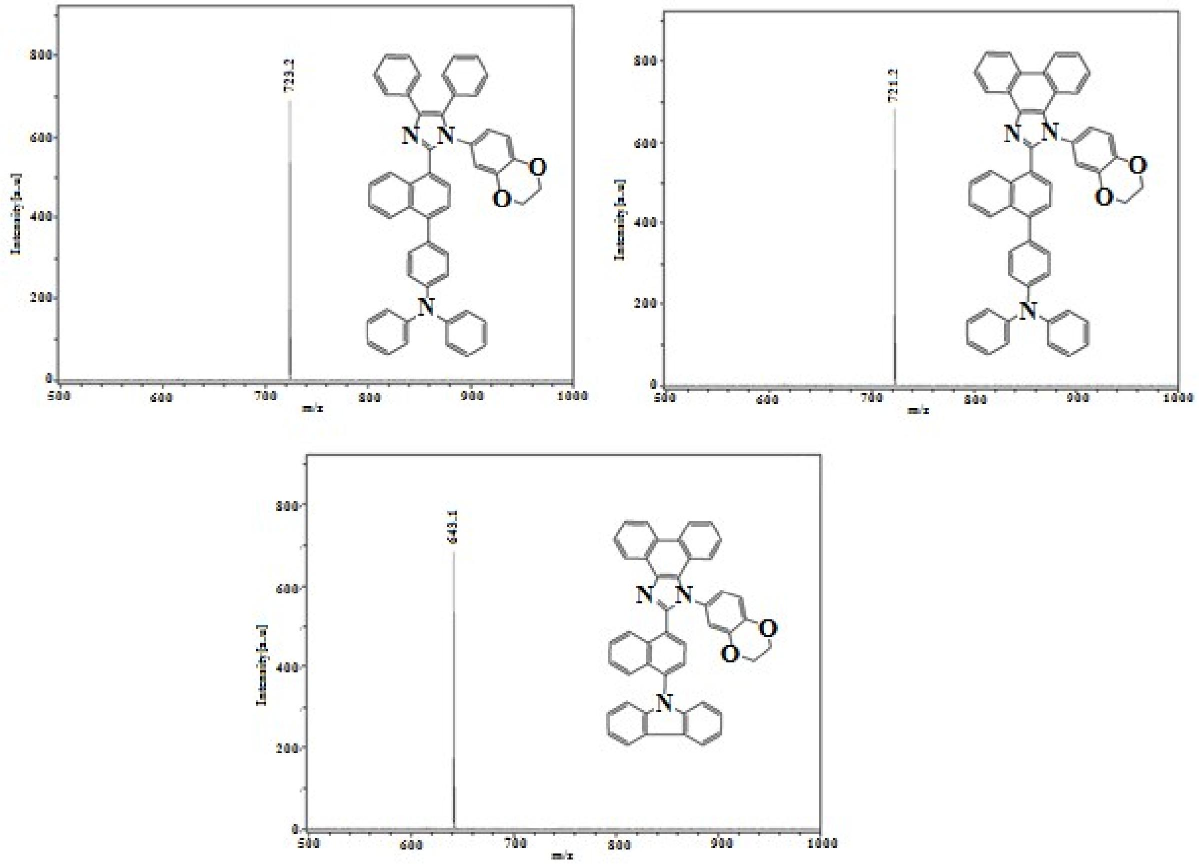

Figure S8. Ground state (S_0) and excited state (S_1) geomentries of DDPB, DBDPA and CDDPI.

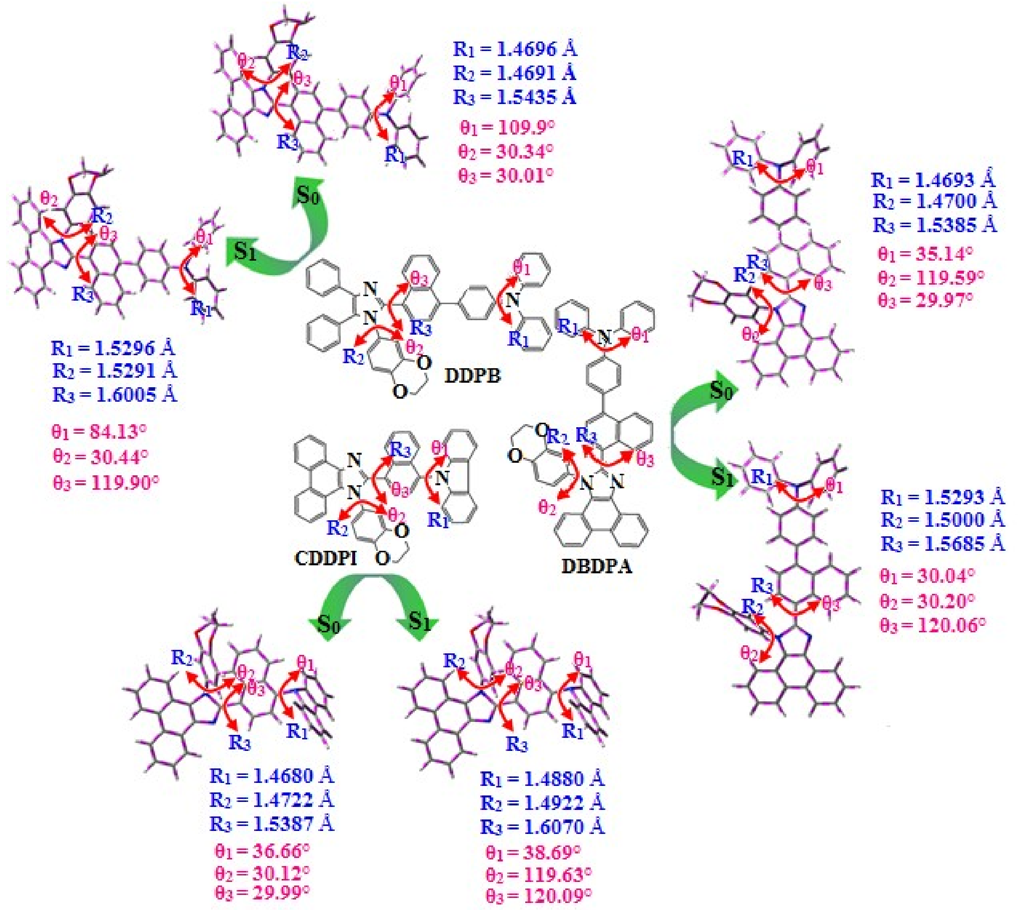

Quasi-equivalent hybridized materials Vs Excellent device performances

The integral overlap of hole-electron (Figure 6 - DDPB; Figure S9 – DBDPA; Figure S10- CDDPI) distribution (S) is a measure of spatial separation of hole and electron. The integral overlap (S) of hole and electron and distance (D) between centroids of hole and electron confirmed the existence of LE and CT states (Table S4-S6). When compared with parent compounds, DDPB, DBDPA and CDDPI has small D and high S value, however, small D and high S of DBDPA on comparison with CDDPI indicates the charge transfer (CT) is higher in percentage for DBDPA isomer. The variation of dipolemoment with respect to S_0 state is outputted which is directly evaluated based on the position of centroid of hole and electron. RMSD of hole or electron characterizes their distribution breadth: RMSD of both electron and hole in DBDPA is higher in X direction, indicates electron and hole distribution is much broader in X direction whereas RMSD of electron in DBDPA (Table S9) is smaller and hole is higher than CDDPI (Table S10). The H intex (half sum of the axis of anistropic density variation distribution) measures the spread of positive and negative regions related to CT. The CT intex, *i.e., t* index (difference between D_{CT} and H intex) is another measure of separation of hole-electron (equations S15 and S16). The D_{CT} of DDPB, DBDPA and CDDPI is calculated to be 0.62, 0.78 and 0.40, respectively (Figure 9: Table 6). For The non-zero *t* of DDPB, DBDPA and CDDPI is negative in all directions which reveal that the overlap of hole and electron is very severe and eign value is greater than 0.97 which supports the hybridization and described in terms of dominant excitation pair in term of 96% of transition. This is further evidenced by Δr intex (equation S1: Tables 3-5) which is average of hole (h^+)-electron (e^-) distance ($d_{h^+-e^-}$) upon excitation which shows the nature of excitation type, LE or CT: valence excitation (LE) is related to short distance (< $d_{h^+-e^-}$) while the larger distance (> $d_{h^+-e^-}$) is related to CT excitation. The triplet exciton is transformed to singlet exciton through RISC process with high energy excited state (hot CT channel) which is beneficial for triplet

exciton conversion in electroluminescence process without any delayed fluorescence (Figure 10). The CT excitons are formed with weak binding energy (E_b) on higher excited states as a result, the exciton utilization (η_s) can be harvested in DDPB, DBDPA and CDDPI like phosphorescent materials. The quasi-equivalent hybridized materials exhibit excellent device performances due to fine modulation in excited states: enhanced LE component and hybridization between LE and CT components results high η_{PL} and high η_s (Table 1).

Figure S9. Hole and particle distribution of DBDPA [S_1–S_5 states: ●-green increasing electron density and ● - blue decreasing electron density (density=transition=n IOp(6/8=3)].

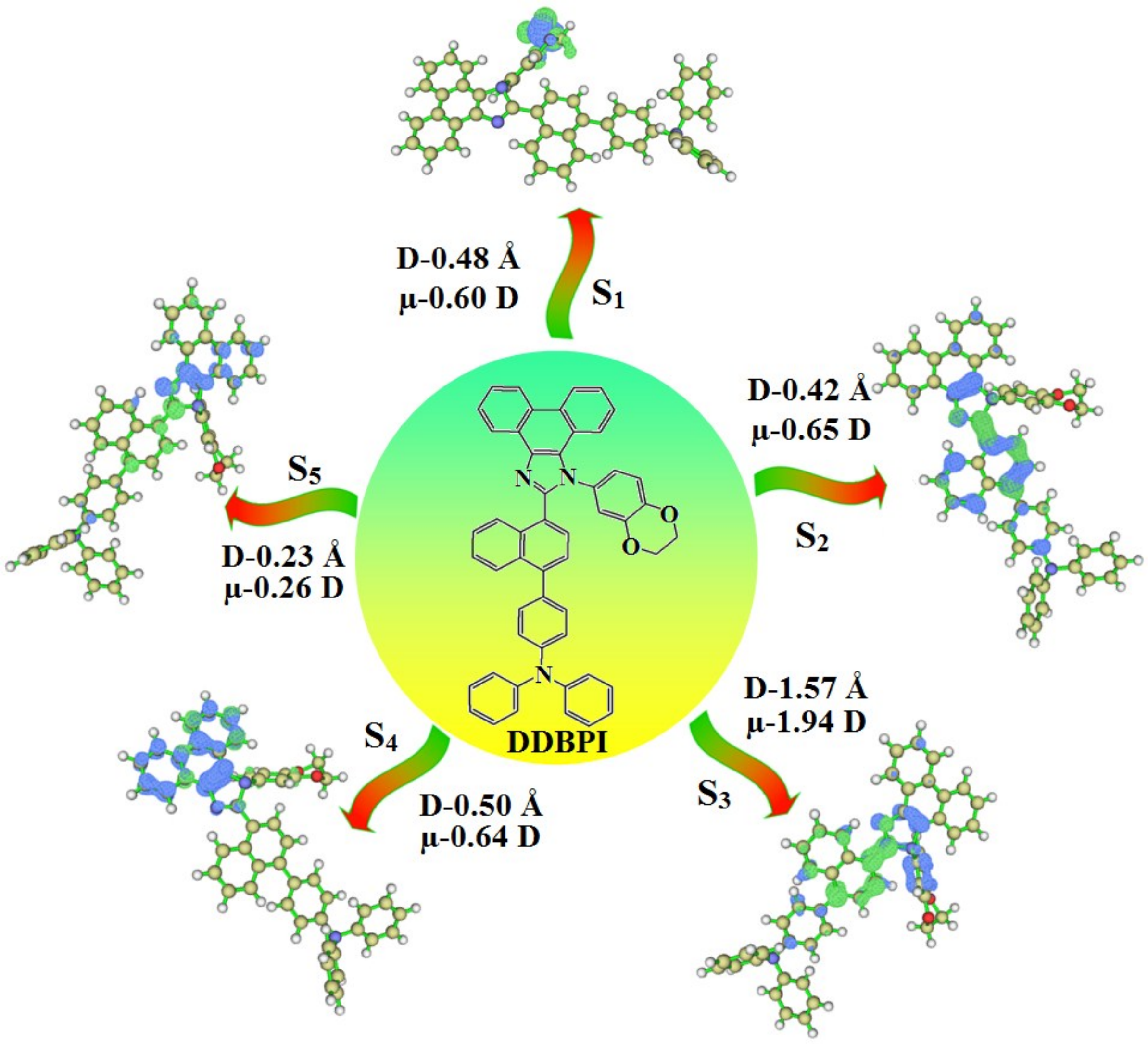

Figure S10. Hole and particle distribution of CDDPI [S_1–S_5 states: ● -green increasing electron density and ● - blue decreasing electron density (density=transition=n IOp(6/8=3)].

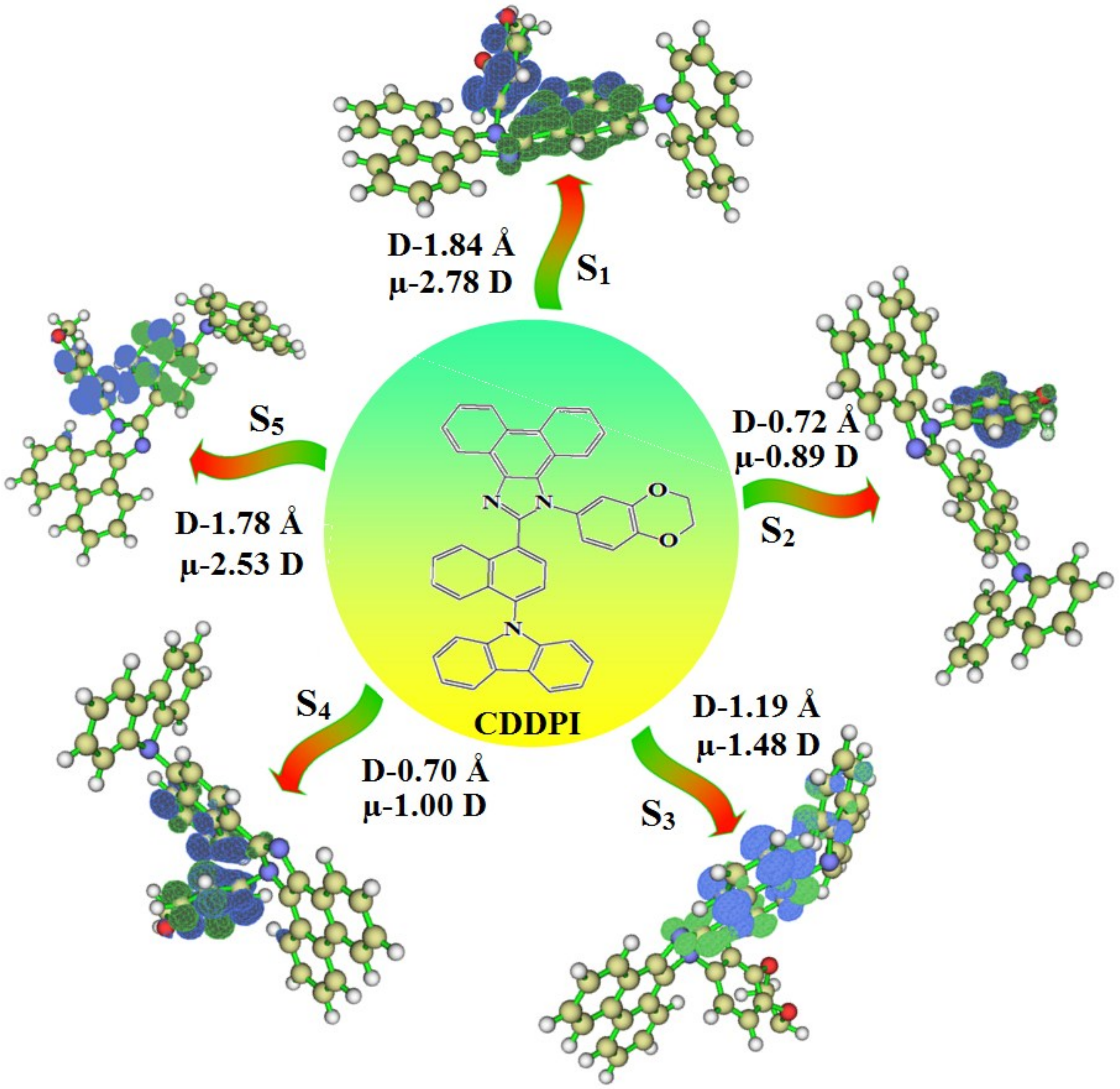

Figure S11. Normalized emission spectra (a and b) and absorption spectra (c and d) of DDPB, DBDPA and CDDPI.

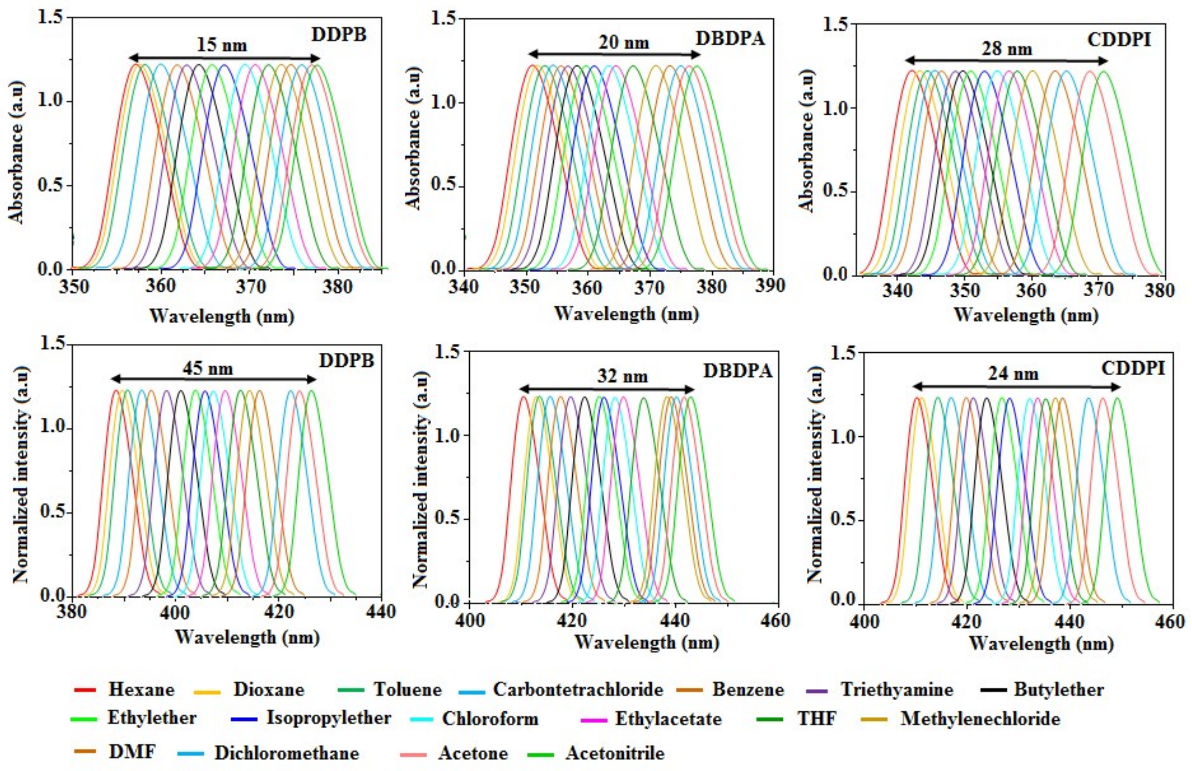

Figure S 12. Highest occupied and lowest unoccupied natural transition orbitals of DDPB

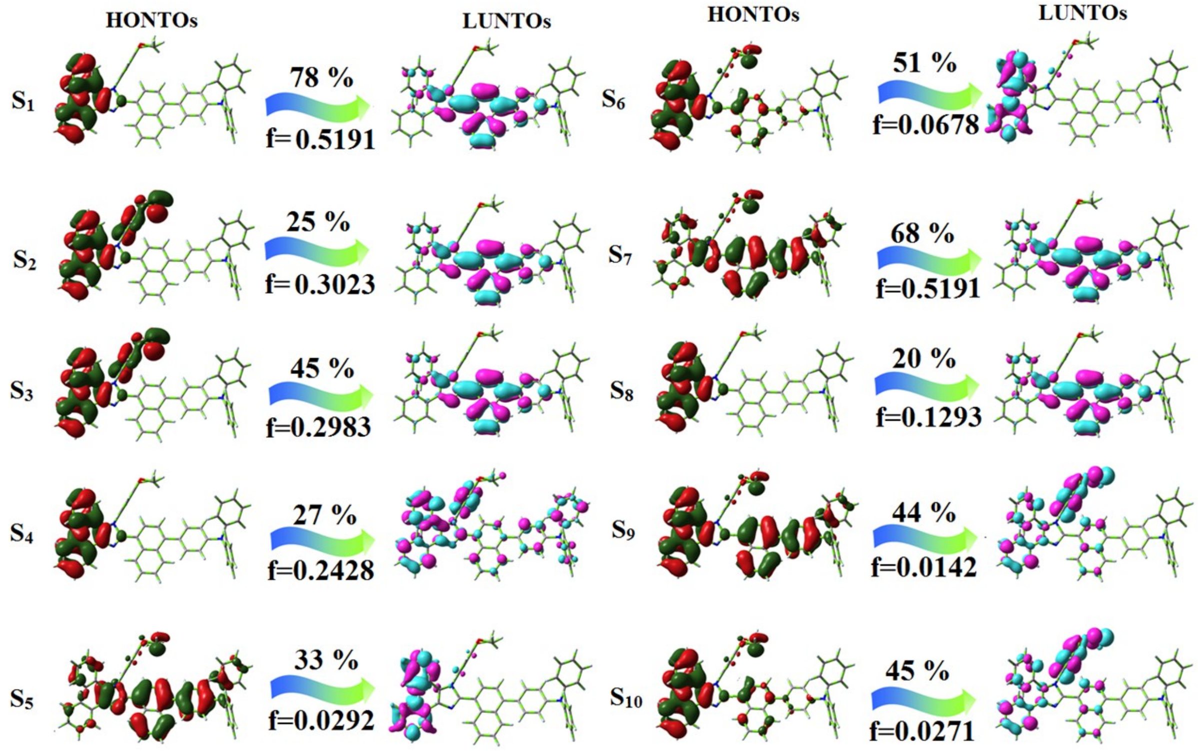

Figure S13. Highest occupied and lowest unoccupied natural transition orbitals of CDDPI

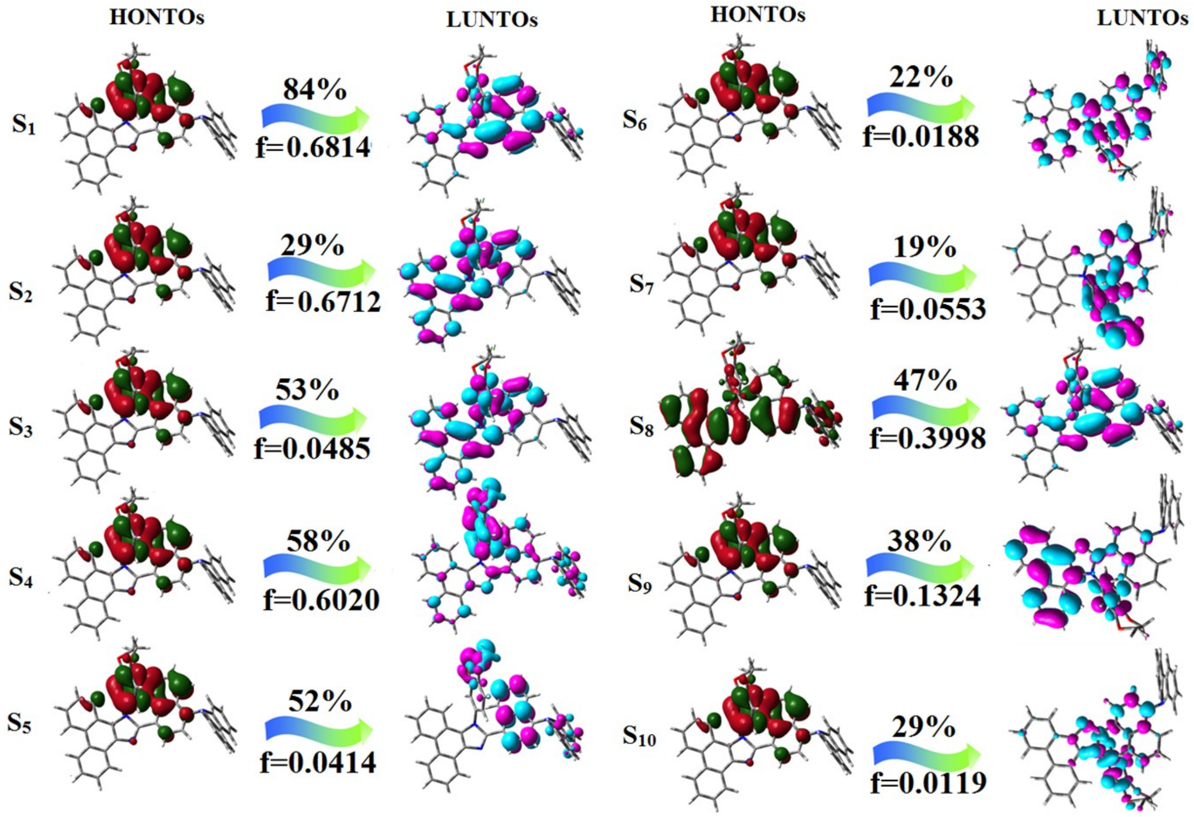

Figure S14. Computed contour plots of transition density matrices (TDM) of DBDPA [density=transition=n /IOp(6/8=3)].

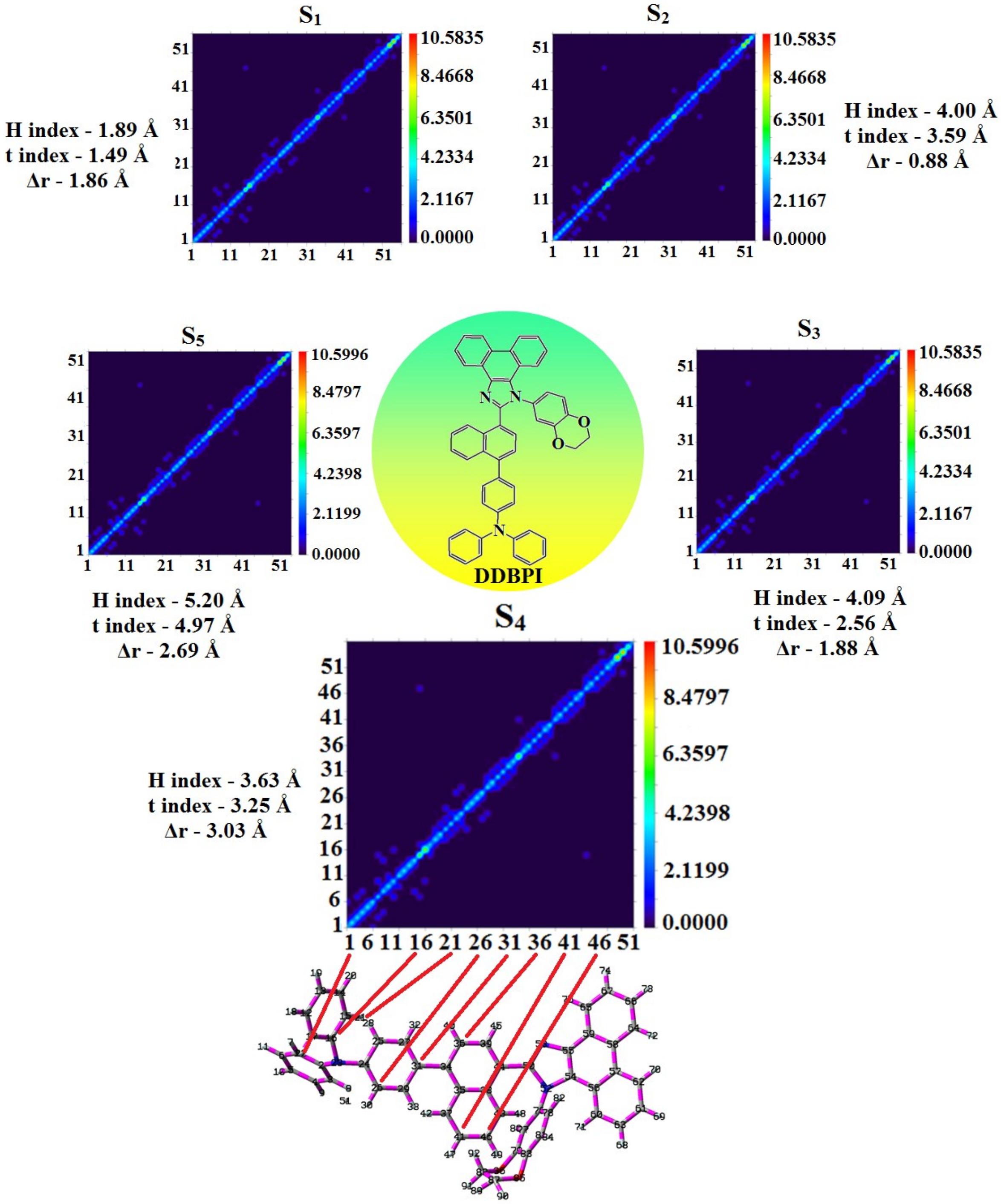

Figure S15. Computed contour plots of transition density matrices (TDM) of CDDPI [density=transition=n /IOp(6/8=3)].

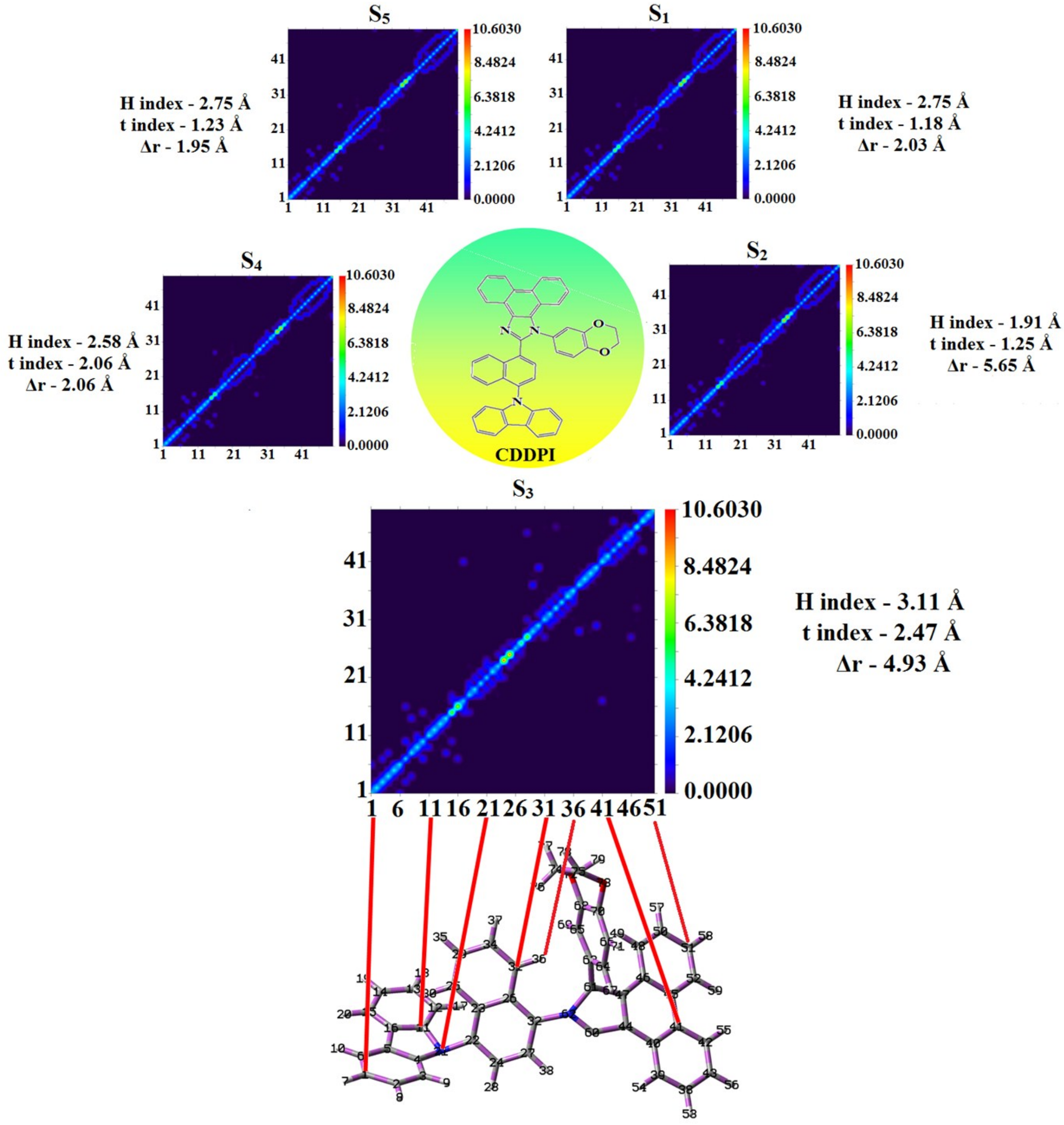

SI-IX: Tables

Table S1. Photophysical properties of DDPB in different solvents

Solvents	ε	n	f(ε,n)	ET(30)	λ_{ab} (nm)	ν_{ab} (cm^{-1})	λ_{flu} (nm)	ν_{flu} (cm^{-1})	ν_{ss} (cm^{-1})	ΔG (kcal/mol)	$\Delta(\Delta G_{hex}-\Delta G_{sol})$ (kcal/mol)	λ (kcal/mol)
Hexane	1.88	1.37	0.000411	32.4	356	27472.53	390	26455.03	1017.501	76.76	0.00	1.34
Dioxane	2.22	1.4226	0.021437	36	357	27397.26	391	26246.72	1150.541	76.36	0.40	1.53
Toluene	2.38	1.494	0.014	33.9	357	27247.96	392	26109.66	1138.296	76.26	0.50	1.63
Carbontetrachloride	2.238	1.46	0.011075	39.1	358	27624.31	391	25974.03	1650.283	76.38	0.38	1.94
Benzene	2.284	1.426	0.026639	34.3	359	27247.96	396	25906.74	1341.221	75.77	0.99	1.91
Triethylamine	3.13	1.432	0.048	31.2	360	27247.96	398	25706.94	1541.016	75.69	1.07	2.20
Butylether	3.57	1.421	0.096	33.4	362	27397.26	403	25641.03	1756.235	75.81	0.95	2.51
Ether	4.27	1.3526	0.164721	34.5	362	27173.91	405	25510.2	1663.709	75.53	1.23	2.79
Isopropylether	3.88	1.368	0.145	33.1	364	27322.4	411	25380.71	1941.694	75.33	1.43	2.78
Chloroform	4.81	1.4459	0.148262	39.1	370	27027.03	412	25316.46	1710.571	75.13	1.63	2.76
Ethyl acetate	6.09	1.4131	0.186569	38.1	366	27397.26	415	25188.92	2208.343	75.39	1.37	3.57
THF	7.52	1.405	0.209634	37.4	369	27247.96	420	23809.52	3438.433	73.00	3.76	5.10
Methylenechloride	7.98	1.427	0.0111	39.1	369	27247.96	421	23640.66	3607.294	72.73	4.03	5.16
Dimethylformamide	8.36	1.413	0.2766	39.8	366	27397.26	422	23474.18	3923.082	72.71	4.05	5.61
Dichloromethane	9.08	1.4242	0.218349	40.7	371	26954.18	435	22988.51	3965.672	72.13	4.63	5.34
Acetone	21.01	1.3588	0.28478	42.2	364	27472.53	430	23255.81	4216.714	72.32	4.44	6.00
Acetonitrile	37.5	1.3442	0.305378	45.6	361	27700.83	432	23148.15	4552.683	72.41	4.35	6.55

Table S2. Photophysical properties of DBDPA in different solvents

Solvents	ε	n	f(ε,n)	ET(30)	λ_{ab} (nm)	ν_{ab} (cm^{-1})	λ_{flu} (nm)	ν_{flu} (cm^{-1})	ν_{ss} (cm^{-1})	ΔG (kcal/mol)	$\Delta(\Delta G_{hex}-\Delta G_{sol})$ (kcal/mol)	λ (kcal/mol)
Hexane	1.88	1.37	0.000411	32.4	359	26881.72	413	24213.08	2668.645	73.03	9.49	3.81
Dioxane	2.22	1.4226	0.021437	36	360	27027.03	415	24096.39	2930.641	73.07	9.45	4.19
Toluene	2.38	1.494	0.014	33.9	361	27247.96	416	26109.66	1138.296	76.26	0.50	1.63
Carbontetrachloride	2.238	1.46	0.011075	39.1	362	26809.65	416	24038.46	2771.19	72.67	9.85	3.96
Benzene	2.284	1.426	0.026639	34.3	363	26737.97	418	23923.44	2814.523	72.41	10.11	4.02
Triethylamine	3.13	1.432	0.048	31.2	364	27247.96	420	25706.94	1541.016	75.69	1.07	2.20
Butylether	3.57	1.421	0.096	33.4	365	27397.26	422	25641.03	1756.235	75.81	0.95	2.51
Ether	4.27	1.3526	0.164721	34.5	365	26881.72	425	23529.41	3352.309	75.53	1.23	2.79
Isopropylether	3.88	1.368	0.145	33.5	366	27322.4	425	25380.71	1941.694	75.33	1.43	2.78
Chloroform	4.81	1.4459	0.148262	39.1	377	26666.67	428	23364.49	3302.181	71.51	11.01	4.72
Ethyl acetate	6.09	1.4131	0.186569	38.1	369	27173.91	429	23310.02	3863.89	72.15	10.37	5.52
THF	7.52	1.405	0.209634	37.4	376	26666.67	431	23201.86	3464.811	71.27	11.25	4.95
Methylenechloride	7.98	1.427	0.0111	39.1	375	27247.96	433	23640.66	3607.294	72.73	4.03	5.16
Dimethylformamide	8.36	1.413	0.2766	39.8	371	27397.26	437	23474.18	3923.082	72.71	4.05	5.61
Dichloromethane	9.08	1.4242	0.218349	40.7	379	26385.22	442	22624.43	3760.790	70.05	12.47	5.38
Acetone	21.01	1.3588	0.28478	42.2	378	26455.03	443	22573.36	3881.663	72.32	4.44	6.00
Acetonitrile	37.5	1.3442	0.305378	45.6	368	27173.91	445	22471.91	4702.003	70.96	11.56	6.72

Table S3. Photophysical properties of CDDPI in different solvents

Solvents	ε	n	f(ε,n)	ET(30)	λ_{ab} (nm)	ν_{ab} (cm^{-1})	λ_{flu} (nm)	ν_{flu} (cm^{-1})	ν_{ss} (cm^{-1})	ΔG (kcal/mol)	$\Delta(\Delta G_{hex}-\Delta G_{sol})$ (kcal/mol)	λ (kcal/mol)
Hexane	1.88	1.37	0.000411	32.4	343	29585.8	405	24691.36	4000.110	92.26	0.00	7.00
Dioxane	2.22	1.4226	0.021437	36.0	344	29411.76	406	24630.54	3000.200	77.24	15.02	6.83
Toluene	2.38	1.494	0.014	33.9	344	27027.03	408	26109.66	1138.296	76.26	10.50	1.63
Carbontetrachloride	2.238	1.46	0.011075	39.1	345	29325.51	408	24509.8	3500.320	76.94	15.32	6.88
Benzene	2.284	1.426	0.026639	34.3	346	29239.77	410	24390.24	4100.240	76.65	15.61	6.93
Triethylamine	3.13	1.432	0.048	31.2	347	28985.51	412	24271.84	3100.117	75.69	11.07	6.74
Butylether	3.57	1.421	0.096	33.4	349	28735.63	413	24213.08	4522.557	75.81	10.95	6.46
Ether	4.27	1.3526	0.164721	34.5	350	28571.43	415	24096.39	4475.043	75.28	75.28	6.40
Isopropylether	3.88	1.368	0.145	33.5	354	28248.59	417	23980.82	4267.772	75.28	75.34	6.10
Chloroform	4.81	1.4459	0.148262	39.1	357	28011.2	418	23923.44	4087.760	74.23	18.03	5.84
Ethyl acetate	6.09	1.4131	0.186569	38.1	351	28490.03	421	23752.97	4009.010	74.67	17.59	6.77
THF	7.52	1.405	0.209634	37.4	358	27932.96	422	23696.68	4005.120	73.79	18.47	6.05
Methylenechloride	7.98	1.427	0.0111	39.1	360	27777.78	423	23640.66	4137.116	73.49	18.77	5.91
Dimethylformamide	8.36	1.413	0.2766	39.8	363	27548.21	425	23529.41	4000.300	73.00	19.26	5.74
Dichloromethane	9.08	1.4242	0.218349	40.7	365	27397.26	429	23310.02	4000.250	72.47	19.79	5.84
Acetone	21.01	1.3588	0.28478	42.2	369	27100.27	431	23201.86	4500.310	71.89	20.37	5.57
Acetonitrile	37.5	1.3442	0.305378	45.6	371	26954.18	433	23094.69	5000.010	71.53	20.73	5.52

Table S4. Hole (H) and electron (E) integral, H-E overlap (S), distance between centroid of H-E (D, Å) and dipolemoment of DDPB

States	Hole integral	Electron integral	Transition density	Overlap of H-E (S)	Centeroid of hole (Å)			Centeroid of electron (Å)			D, Å	Dipole moment μ(D)
					X	Y	Z	X	Y	Z		
S_1	0.7161	0.7273	0.0034	0.3890	-6.9883	-0.4496	0.6447	-6.5225	0.7612	0.3192	0.65	0.94
S_2	0.7064	0.5052	0.0020	0.1639	-0. 4587	3.9062	-0.3817	-0.3372	4.3370	0.3716	0.64	0.73
S_3	0.7931	0.7109	0.0061	0.2157	-6.5118	-0.4759	-0.5327	-3.0626	-0.7217	-0.4654	3.39	5.11
S_4	0.7470	0.6387	0.0029	0.2894	-6.6082	-0.4679	0.5635	-5.4237	-1.4079	0.1004	1.58	2.07
S_5	0.7548	0.5102	-0.0848	0.2936	-6.9883	-0.4496	0.6447	-6.5705	-0.6565	0.4407	0.50	0.61
S_6	0.7414	0.6967	-0.0513	0.3085	-6.9883	-0.4496	0.6447	-7.1431	-0.2747	0.8113	0.28	0.33
S_7	0.8564	0.5443	-0.0045	0.4401	-0.2509	-1.2688	-0.5887	-0.7854	-1.1902	-0.8332	0.59	0.87
S_8	0.6610	0.5988	-0.0029	0.2518	-5.0814	-0.2935	0.3198	-1.7848	-0.9448	-0.5320	3.46	3.94
S_9	0.7348	0.4309	-0.0011	0.1159	-6.2870	0.1817	0.5196	-2.2602	1.9324	-0.1721	4.60	5.79
S_{10}	0.6414	0.4894	-0.0005	0.2146	-4.5372	0.0649	0.2306	-1.6024	-1.7581	-0.5466	3.14	3.36

Table S5. Hole (H) and electron (E) integral, H-E overlap(S), distance between centroid of H-E (D, Å) and charge density difference of DBDPA

States	Integral of hole	Integral of electron	Integral of transition density	Integral of overlap of H-E (S)	Centeroid of hole (Å)			Centeroid of electron (Å)			D, Å	Dipole moment μ(D)
					X	Y	Z	X	Y	Z		
S_1	0.7879	0.5335	0.0150	0.1531	2.3028	6.1516	-.6225	2.4798	5.9065	.2411	0.48	0.60
S_2	0.8907	0.7261	-0.0071	0.4808	0.9822	1.0924	0.0448	0.5602	1.1528	0.0087	0.42	0.65
S_3	0.7115	0.5896	-0.0165	0.2447	2.0350	0.3737	0.0237	0.6464	1.1265	0.0094	1.57	1.94
S_4	0.7837	0.5709	-0.0002	0.4580	5.8429	0.9325	0.1980	5.9632	0.4438	0.1728	0.50	0.64
S_5	0.6690	0.5199	-0.0073	0.3438	2.7184	0.8338	0.1256	2.5246	0.9736	0.1155	0.23	0.26
S_6	0.8299	0.6005	0.0057	0.4905	0.4087	1.7564	0.1938	0.2035	1.2839	-.0543	0.78	1.06
S_7	0.6837	0.5003	-0.0039	0.2757	3.5455	0.3334	0.2625	4.0030	0.4280	0.3569	0.47	0.53
S_8	0.4935	0.4392	-0.0018	0.1375	1.8727	2.0661	0.4764	0.5439	1.5065	0.1163	2.50	2.20
S_9	0.6026	0.4309	-0.0022	0.3153	1.3698	1.0836	0.1093	2.1424	1.2043	0.1359	0.78	0.76
S_{10}	0.6519	0.4873	0.0005	0.3368	2.1677	0.4557	0.1877	1.9819	0.7336	0.0254	0.37	0.40

Table S6. Hole (H) and electron (E) integral, H-E overlap (S), distance between centroid of H-E (D, Å) and dipolemoment of CDDPI

State	Hole integral	Electron integral	Integral of transition density	Integral overlap of hole & electron (S)	Centroid of hole (Å)			Centroid of electron (Å)			D (Å)	μ (a.u)
					x	y	z	x	y	z		
S_1	0.8562	0.7414	-0.0007	0.3130	-0.5339	1.8497	-0.0838	0.7540	0.5396	-0.2179	1.84	2.78
S_2	0.7586	0.5478	0.0019	0.1522	-1.4914	4.7891	-0.4926	-1.7149	5.4561	-0.3291	0.72	0.89
S_3	0.7217	0.5855	-0.0910	0.2611	-2.3818	-2.3538	0.4280	-2.1877	-1.2282	0.0635	1.19	1.48
S_4	0.8549	0.6531	0.0249	0.3114	-0.5339	1.8497	-0.0838	-1.0275	1.8454	0.4158	0.70	1.00
S_5	0.7871	0.5941	0.0112	0.2409	-0.5339	1.8497	-0.0838	1.0806	1.1170	-0.3060	1.78	2.33
S_6	0.7559	0.5249	0.0076	0.1413	-0.5339	1.8497	-0.0838	-1.5030	-0.3718	-0.3169	2.43	2.94
S_7	0.7930	0.4281	-0.0088	0.1473	-0.7078	1.4527	-0.0966	-1.2366	3.0410	-0.2581	1.68	1.94
S_8	0.8192	0.6409	-0.0082	0.3802	-0.5761	0.2409	-0.1040	-1.2439	-0.1934	-0.2482	0.80	1.11
S_9	0.8279	0.6464	0.0001	0.2326	-0.7226	1.4189	-0.0977	-2.7702	-0.6578	-0.2696	2.92	4.07
S_{10}	0.7912	0.3910	-0.0059	0.0704	-0.5339	1.8497	-0.0838	-2.1763	3.0730	-0.1858	2.05	2.29

Table S7: Computed RMSD of electron and hole, H index and t index of singlet states of DDPB

State	Electron RMSD				Hole RMSD				H index				t index			
	x	y	z	total	x	y	z	total	x	y	z	Total	x	y	z	Total
S_1	1.673	1.985	1.059	2.804	1.373	2.149	1.047	2.757	1.523	2.067	1.053	2.775	-1.058	-1.756	-0.728	2.175
S_2	1.864	2.012	1.073	2.945	1.702	1.835	0.744	2.611	1.783	1.923	0.908	2.776	-1.676	-1.293	-0.898	2.299
S_3	3.219	1.811	1.132	3.863	2.290	2.122	1.082	3.304	2.754	1.966	1.107	3.561	0.695	-1.721	-0.109	1.859
S_4	3.687	2.177	1.110	4.423	2.188	2.136	1.076	3.241	2.938	2.156	1.093	3.804	-1.753	-1.216	-0.630	2.225
S_5	2.008	2.207	1.053	3.164	1.373	2.149	1.047	2.757	1.691	2.178	1.050	2.950	-1.273	-1.971	-0.846	2.494
S_6	1.550	2.350	1.097	3.021	1.373	2.149	1.047	2.757	1.462	2.250	1.072	2.889	-1.307	-2.075	-0.905	2.614
S_7	2.881	1.526	0.825	3.363	3.885	1.801	1.002	4.398	3.383	1.663	0.913	3.879	-2.848	-1.585	-0.669	3.328
S_8	3.394	2.098	1.027	4.120	3.562	2.372	1.150	4.432	3.478	2.235	1.088	4.275	-0.181	-1.584	-0.237	1.612
S_9	1.780	2.008	1.143	2.916	2.280	2.283	1.075	3.401	2.030	2.145	1.109	3.155	1.997	-0.031	-0.417	2.040
S_{10}	3.326	2.294	1.030	4.169	3.410	2.466	1.132	4.357	3.368	2.380	1.081	4.263	-0.433	-1.557	-0.304	1.644

Table S8: Computed RMSD of electron and hole, H index and t index of singlet states of DBDPA

State	Electron RMSD				Hole RMSD				H index				t index			
	**x**	**y**	**z**	**total**	**x**	**y**	**z**	**total**	**x**	**y**	**z**	**Total**	**x**	**y**	**z**	**Total**
**S_1**	1.046	1.369	0.773	1.888	0.999	1.476	0.692	1.911	1.022	1.422	0.732	1.899	-0.845	-1.177	-0.351	1.491
**S_2**	3.023	1.535	0.770	3.477	4.110	1.743	0.809	4.537	3.567	1.639	0.789	4.004	-3.145	-1.579	-0.753	3.598
**S_3**	3.094	1.578	0.771	3.558	3.911	2.353	0.838	4.641	3.503	1.965	0.805	4.096	-2.114	-1.213	-0.790	2.562
**S_4**	2.824	2.309	0.766	3.728	2.673	2.209	0.760	3.550	2.749	2.259	0.763	3.639	-2.629	-1.770	-0.738	3.254
**S_5**	4.491	1.832	0.769	4.911	5.072	1.967	0.789	5.497	4.782	1.900	0.779	5.204	-4.588	-1.760	-0.769	4.974
**S_6**	2.477	1.779	0.755	3.142	2.237	1.884	0.759	3.021	2.357	1.831	0.757	3.079	-1.745	-1.359	-0.617	2.296
**S_7**	2.952	1.594	0.864	3.464	3.672	1.977	0.848	4.256	3.312	1.786	0.856	3.859	-2.854	-1.691	-0.761	3.404
**S_8**	3.184	1.778	0.765	3.726	2.825	1.646	0.592	3.322	3.004	1.712	0.679	3.524	-0.587	-1.152	-0.319	1.332
**S_9**	3.221	2.354	0.909	4.092	3.974	2.540	0.926	4.806	3.597	2.447	0.918	4.446	-2.825	-2.326	-0.891	3.766
**S_{10}**	4.988	1.923	0.848	5.413	4.640	2.248	0.833	5.223	4.814	2.085	0.840	5.313	-4.628	-1.807	-0.678	5.015

Table S9: Computed RMSD of electron and hole, H index and t index singlet states of CDDPI

State	Electron RMSD				Hole RMSD				H index				t index			
	x	y	z	total	x	y	z	total	x	y	z	Total	x	y	z	Total
S_1	2.132	1.834	0.968	2.974	1.974	1.221	1.068	2.555	2.053	1.527	1.018	2.754	-0.765	-0.217	-0.884	1.189
S_2	1.055	1.342	1.028	1.993	0.846	1.498	0.697	1.856	0.951	1.420	0.863	1.914	-0.727	-0.753	-0.699	1.259
S_3	2.973	1.523	0.761	3.426	2.218	1.558	0.793	2.824	2.596	1.540	0.777	3.116	-2.401	-0.415	-0.412	2.472
S_4	1.689	1.695	1.115	2.640	1.974	1.221	1.068	2.555	1.832	1.458	1.091	2.583	-1.338	-1.454	-0.592	2.062
S_5	2.059	1.917	1.014	2.991	1.974	1.221	1.068	2.555	2.016	1.569	1.041	2.759	-0.402	-0.837	-0.819	1.238
S_6	3.089	2.484	0.817	4.047	1.974	1.221	1.068	2.555	2.531	1.853	0.942	3.275	-1.562	0.369	-0.709	1.755
S_7	1.909	3.017	0.993	3.705	2.328	1.669	1.052	3.052	2.118	2.343	1.022	3.320	-1.590	-0.755	-0.861	1.959
S_8	2.994	2.621	0.840	4.067	3.126	2.042	1.022	3.872	3.060	2.332	0.931	3.958	-2.392	-1.897	-0.787	3.153
S_9	3.149	2.352	0.768	4.005	2.355	1.698	1.051	3.088	2.752	2.025	0.909	3.536	-0.704	0.052	-0.737	1.021
S_{10}	1.981	3.255	1.079	3.960	1.974	1.221	1.068	2.555	1.977	2.238	1.073	3.173	-0.335	-1.015	-0.971	1.444

Table S10. Percentage transition of LE and CT of DDPB, DBDPA and CDDPI

**% transition**	**DDPB**		**DBDPA**		**CDDPI**	
	**Singlet (S_1-S_{10})**	**Triplet (T_1-T_{10})**	**Singlet (S_1-S_{10})**	**Triplet (T_1-T_{10})**	**Singlet (S1-S10)**	**Triplet (T1-T10)**
% CT	80	75	90	80	62	72
% LE	20	25	10	20	38	28
